# Preterm Birth and Perinatal Mortality During the COVID‐19 Pandemic Period: A Systematic Review and Meta‐Analysis

**DOI:** 10.1111/jpc.70403

**Published:** 2026-05-02

**Authors:** S. R. Peart, R. Haj‐Yahya, M. Nugent, O. Ganbold, L. Harbinson, Cheong JLY, B. J. Manley, C. L. Whitehead

**Affiliations:** ^1^ Neonatal Services The Royal Women's Hospital Melbourne Parkville Australia; ^2^ Clinical Sciences Murdoch Children's Research Institute Parkville Australia; ^3^ Department of Obstetrics, Gynaecology and Newborn Health The University of Melbourne Parkville Australia; ^4^ Department of Maternal Fetal Medicine The Royal Women's Hospital Melbourne Parkville Australia; ^5^ Department of Paediatrics Western Health Melbourne Australia; ^6^ Department of Medicine, Melbourne Medical School University of Melbourne Parkville Australia; ^7^ Department of Paediatrics Mercy Hospital for Women Melbourne Australia

**Keywords:** coronavirus, COVID‐19, meta‐analysis, perinatal mortality, preterm birth, SARS‐CoV‐2, stillbirth, systematic review

## Abstract

**Background:**

Preterm birth rates may have been affected during the COVID‐19 pandemic but the impact of this on perinatal morbidity is unknown.

**Aim:**

To review the impact of the COVID‐19 pandemic on rates of preterm birth and perinatal mortality.

**Methods:**

Medline, Embase, and online pre‐prints were searched from Jan 2020 to Oct 2022. Case–control, cohort studies and reports comparing rates of preterm birth, stillbirth and neonatal death before and during the COVID‐19 pandemic period were included. The pooled odds ratio (OR) for preterm birth, stillbirth and neonatal death was calculated using a random effects model. The primary outcome was the rate of preterm birth, stillbirth and neonatal death in the pre‐pandemic and pandemic periods.

**Results:**

100 studies were included. Compared with pre‐pandemic periods, there was a decrease in preterm births during the pandemic period: OR 0.95 (95% CI 0.94–0.97) *I* ^*2*^ = 0.93, with the greatest reduction for births < 28 weeks' gestation in high‐income countries: OR 0.92 (95% CI 0.88–0.96), *I* ^*2*^ = 0.46. There was a reduction in neonatal deaths in high‐income countries: OR 0.78 (95% CI 0.64–0.95), *I* ^*2*^ = 0.4. In low‐ and middle‐income countries the stillbirth rate increased during the pandemic compared with the pre‐pandemic period: OR 1.18 (95% CI 1.02–1.36), *I* ^*2*^ = 0.86.

**Conclusion:**

The COVID‐19 pandemic was associated with a reduction in preterm births and neonatal deaths. Further research is needed to investigate the mechanisms underlying these findings. Stillbirth rates increased in low‐ and middle‐income countries where access to healthcare may have been restricted and strategies to address this in future pandemics are warranted.

## Keypoints

- What is already known on this topic
  ○ Preterm birth is the leading cause of infant mortality worldwide with minimal reduction in preterm birth rates in the last 20 years.
  ○ There have been conflicting reports regarding preterm birth rates in response to the COVID‐19 pandemic mitigation strategies with the impact on preterm birth and perinatal mortality at a global level unknown.
- What this paper adds
  ○ This systematic review is the largest to focus on both preterm birth rates and perinatal mortality globally.
  ○ It found a reduction in preterm birth rates with the greatest impact in preterm birth < 28 weeks' gestation.
  ○ In high‐income countries this corresponded to a reduction in neonatal deaths, without an increase in stillbirths, whilst in low‐ and middle‐income countries, the stillbirth rate increased.

**Abbreviations**
CI: Confidence interval
COVID‐19: Coronavirus disease 2019
GRSI: Government Response Stringency Index
HIC: High‐income country
LMIC: Low‐ and middle‐income countries
OR: Odds ratio
PTB: Preterm Birth
SARS‐CoV‐2: severe acute respiratory syndrome coronavirus 2
WHO: World Health Organization

## 1 Introduction

Preterm birth (< 37 weeks' gestation, PTB) affects 10% of livebirths and is the leading global cause of early childhood morbidity and mortality [1, 2]. PTB is heterogeneous, the multifactorial aetiology of which is not fully understood but includes medical, environmental and sociodemographic influences [3]. Children born preterm are at increased risk of longer‐term complications including neurodevelopmental delay, disability and early adult death compared with those born at term [4]. Rates of PTB, particularly in high‐income countries, are increasing [5], and there is a global effort aimed at developing interventions that reduce the incidence and burden of complications from PTB [6]. However, effective interventions are limited and new opportunities to improve our knowledge and understanding of PTB are urgently sought.

In March 2020, the World Health Organization (WHO) declared COVID‐19 a worldwide pandemic, with mitigation measures instituted in many countries to reduce transmission of the SARS‐CoV‐2 virus. Mitigation measures varied between countries and regions but included social distancing (maintaining a minimum distance between yourself and others), stay at home orders or restrictions on reasons to leave the home (‘lockdowns’), and a greater adherence to hygiene practices. Such measures significantly affected the way maternity care was delivered. In many countries there was reduced access to healthcare providers during pregnancy and labour [7], raising concern for a potential increase in adverse pregnancy outcomes, including stillbirth [8]. Additionally, infection with SARS‐CoV‐2 during pregnancy was associated with an increased risk of PTB and stillbirth [9, 10].

Early reports arose of a reduction in PTB shortly after mitigation measures were implemented [11]. It was unclear if this was due to a reduction in spontaneous PTB or a reduction in medically indicated PTB due to a failure to diagnose foetal growth restriction and pre‐eclampsia secondary to reduced access to healthcare. Initial reports of reduced PTB rates came at the expense of increased stillbirths [12]. However, such effects appeared to vary considerably between geographical locations and the stringency of mitigation strategies implemented [13]. It remains unclear what impact the pandemic had on PTB, stillbirth, and neonatal deaths at a global level. There have been several systematic reviews and meta‐analyses conducted to examine the impact of pandemic restrictions on perinatal outcomes globally [13, 14, 15, 16, 17, 18]. However, comparison between studies was limited as reviews were published during the pandemic and conflicting regional reports were emerging. Concerns about small changes in perinatal outcomes due to methodological challenges in single reports limited the applicability of results at a global population level.

### 1.1 Objective

To overcome this, we have performed the largest global systematic review of perinatal outcomes during the period of the COVID‐19 pandemic, comparing periods of mitigation measures with pre‐pandemic periods to determine the impact on PTB rates.

## 2 Methods

This review was conducted according to PRISMA guidelines [19] and the study protocol was prospectively registered with PROSPERO (#CRD42020221200).

## 3 Search Strategy and Selection Criteria

We electronically searched Medline, Embase and MedRxiv from 1st January 2020 to 4th October 2022, without language or geographic restriction (Figure S1). Peer‐reviewed publications and pre‐prints of case–control, cohort and primary reports were eligible for inclusion. Editorials (without primary data), systematic reviews, commentaries, news articles and studies only including SARS‐CoV‐2‐infected pregnant women were excluded. Two of three independent reviewers (SP, RH‐Y, MN) screened all abstracts and extracted data from the selected full texts using a standardised form in Covidence (Veritas Health Innovation, Melbourne, Australia). Any disagreements between the reviewers were resolved by consensus or a fourth reviewer (CLW).

## 4 Outcomes

Primary outcomes included rates of PTB, stillbirth, and neonatal death as defined according to the primary data source. The effect of economic status and the stringency of mitigation measures on the primary outcomes was assessed. Planned subgroup analysis included rates of PTB by gestational age subgroups: moderate/late preterm (32–36 completed weeks' gestation), very preterm (28–31 weeks'), and extremely preterm (< 28 weeks'), the mode of birth (caesarean or vaginal), and indication for delivery (spontaneous or medically indicated).

## 5 Data Extraction and Risk of Bias Assessment

Data extraction was performed using a standardised form designed by two investigators (SP, RH‐Y). Information collected included: study design, setting (national, regional/multisite, and single centre), geographic location, pre‐pandemic and pandemic sampling periods (defined respectively as the period prior, during and after mitigation measures were implemented), and study population. The maximum pandemic mitigation response measures implemented during the study reporting period were recorded according to the University of Oxford's Government Response Stringency Index (GRSI) [20]. Countries, including as part of international, national or single site studies, with a stringency index of ≥ 80 were classified as high stringency (reflecting a stricter response in mitigation measures), while those with lower indices were classified as low stringency. Economic status as either a high‐income country (HIC) or low‐ and middle‐income countries (LMIC) was defined according to the World Bank [21]. The quality of the evidence from each study was scored according to the relevant Newcastle‐Ottawa Scale [22].

## 6 Data Synthesis and Analysis

We conducted quantitative meta‐analyses when data from multiple studies were available. For each outcome before and during mitigation strategies, a summary odds ratio (OR) with a 95% confidence interval (CI) was calculated using a random effects model with the DerSimonian‐Laird method. Subgroup analyses were: (a) HIC versus LMIC; (b) gestational age at birth; and (c) lockdown stringency level. Between‐study heterogeneity was assessed using the *I* ^*2*^ statistic, with interpretation thresholds as follows: small (25%), moderate (50%), and large (75%) [23].

If the outcomes were pooled from more than three studies, then publication bias was assessed using visual examination of funnel plot asymmetry and Begg's and Egger's test. An asymmetrical funnel plot or a *p*‐value < 0.1 was considered to indicate the presence of publication bias. All the statistical analyses were performed using Stata IC version 18 (StataCorp, College Station, TX).

## 7 Results

### 7.1 Study Selection and Study Characteristics

A total of 5655 studies were identified, and 100 met inclusion criteria for data extraction (Figure S1). These studies represent populations from 41 countries; 13 studies were conducted in LMIC and 28 in HIC. Sixty studies were multicentre: 3 were international, 30 regional and 27 national. Eighty‐seven studies reported on PTB rates over 95 observational periods (total 2 562 484 births); 63 studies reported stillbirth rates over 66 observational periods (300 778 births); and 19 studies reported neonatal death rates over 19 observational periods (12 300 infants). Table S1 shows the characteristics of the included studies.

### 7.2 Risk of Bias

**FIGURE 1:**
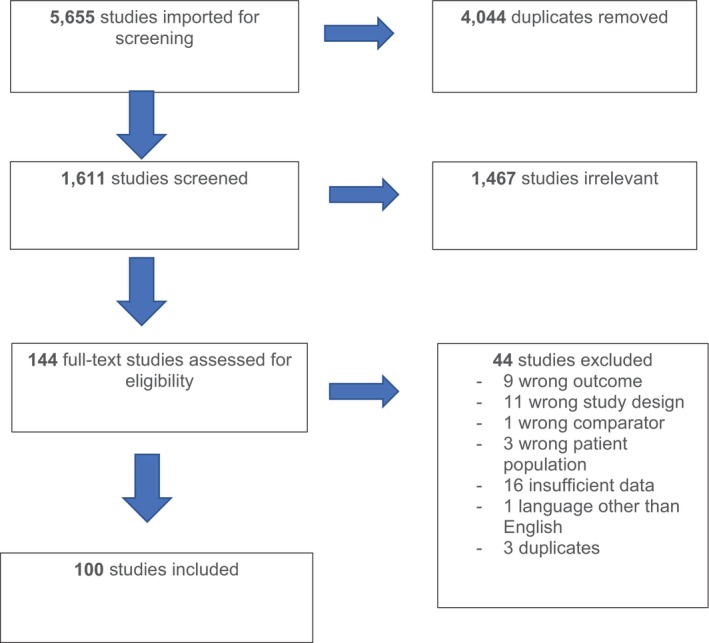
PRISMA flow diagram.

Most studies were of high methodological rigour (score 7–9) on the Newcastle Ottawa Scale (Table S2). The major differentiating factor between studies was cohort comparability. There was heterogeneity in the periods compared, with some studies not accounting for natural temporal trends observed in birth rates Figure 1.

### 7.3 Preterm Birth

**FIGURE 2:**
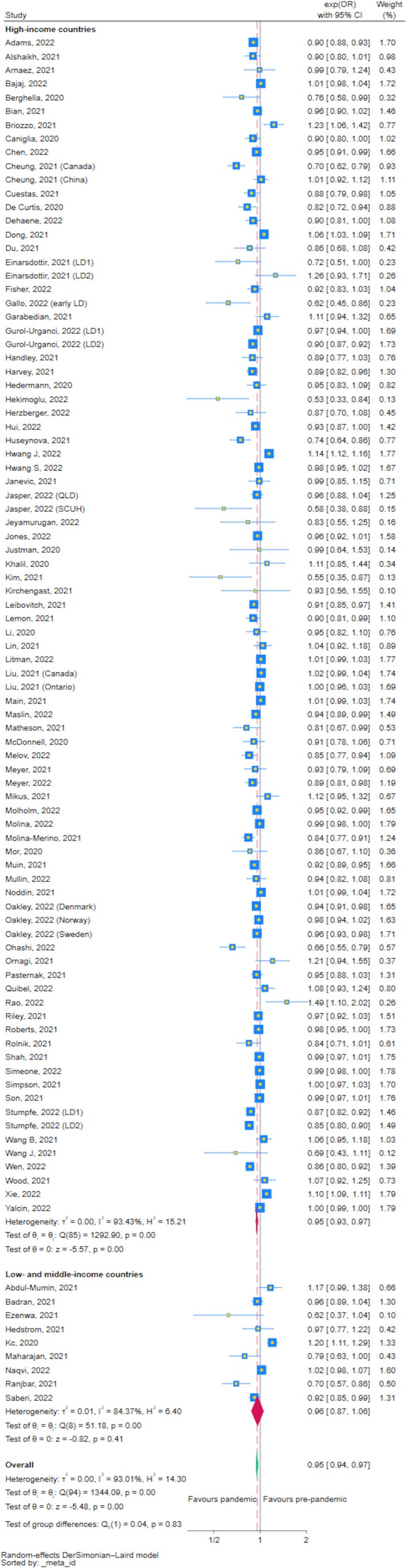
Rates of preterm birth in pre‐pandemic and pandemic periods according to World Bank Classification.

There was a decrease in PTB rates during the pandemic (87 studies, *n* = 561 733 births) compared with pre‐pandemic cohorts (*n* = 2 000 751 births): pooled OR 0.95 (95% CI 0.94–0.97), *I* ^*2*^ = 0.93 (Figure 2). There was evidence of publication bias for this outcome (Egger's test *p* < 0.001) (Figure S2). Subgroup analysis according to gestational age revealed the greatest reduction in extremely PTB during the pandemic (*n* = 10 198 births) compared with pre‐pandemic periods (*n* = 48 442 births): OR 0.92 (95% CI 0.88–0.96), *I* ^*2*^ = 0.46 (Figure S3a). There was also a reduction in moderate/late PTB: OR 0.95 (95% CI 0.93–0.97), *I* ^*2*^ = 0.93 (Figure S3c). Evidence was weak for any effect on very PTB rates: OR 0.96 [95% CI 0.90–1.03], *I* ^*2*^ = 0.93 (Figure S3b).

Sub‐analysis found the reduction in PTB was driven by HICs (OR 0.95 [95% CI 0.93–0.97], *I* ^*2*^ = 0.93; Figure 2), rather than in LMICs (OR 0.96 [95% CI 0.87–1.06], *I* ^*2*^ = 0.84). Meta‐analysis did not demonstrate a difference in PTB rates according to the stringency of mitigation measures implemented (*p* = 0.33; Figure S4).

Twenty‐two studies reported on the indication for PTB over 23 observational periods (*n* = 1 078 438 births). There was a decrease in spontaneous PTB rates during the pandemic compared with the pre‐pandemic period (OR 0.95 (95% CI 0.93–0.98), *I* ^*2*^ = 0.5, Figure S5a) in 20 studies covering 21 observational periods. Sub‐analysis demonstrated a lower spontaneous PTB rate in the setting of low stringency mitigation measures: OR 0.93 [95% CI 0.90–0.97] (Figure S5b). There was no difference in rates of medically indicated PTB during the pandemic in 20 studies covering 22 observational periods: OR 0.97 (95% CI 0.91–1.03), *I* ^*2*^ = 0.97 (Figure S5c).

Low birth weight may represent both PTB where gestation was unknown (in some LMIC) and potential foetal growth restriction. Although there was little difference in rates of low birth weight (< 2500 g) between the pandemic and pre‐pandemic periods (33 studies, 34 observational periods, *n* = 540 589 births): OR 0.97 (95% CI 0.94–1.0), *I* ^*2*^ = 0.92 (Figure S6a), very low birth weight (< 1500 g) births were lower during the pandemic period compared with the pre‐pandemic period: OR 0.90 (95% CI 0.83–0.98), *I* ^*2*^ = 0.73 (Figure S6b).

### 7.4 Stillbirth

**FIGURE 3:**
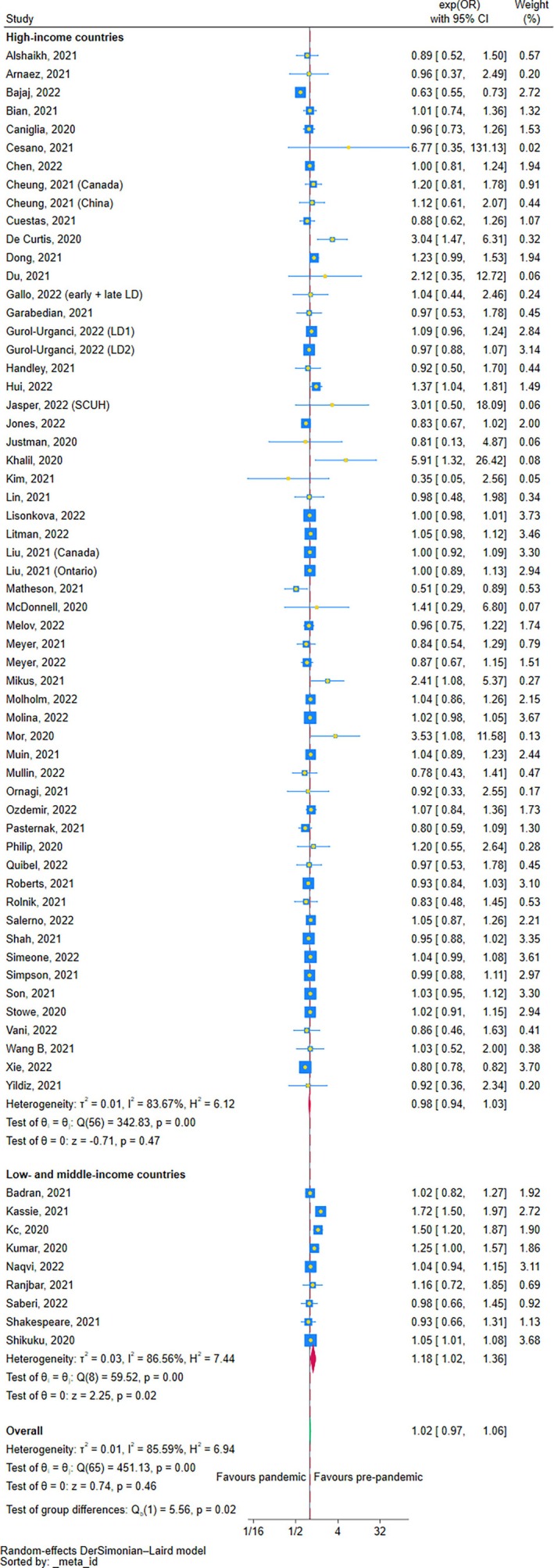
Rates of stillbirth in pre‐pandemic and pandemic periods according to World Bank Classification.

Overall, stillbirth rates were similar during the pandemic period compared with the pre‐pandemic period in 63 studies covering 66 observational periods: OR 1.02 (95% CI 0.97–1.1), *I* ^*2*^ = 0.86. However, there was an increase in stillbirth rates in LMIC: OR 1.18 (95% CI 1.02–1.36), *I* ^*2*^ = 0.86 (Figure 3). No effect of stringency of mitigation measures on rates of stillbirth was observed (*p* = 0.16) (Figure S7).

### 7.5 Neonatal Death

**FIGURE 4:**
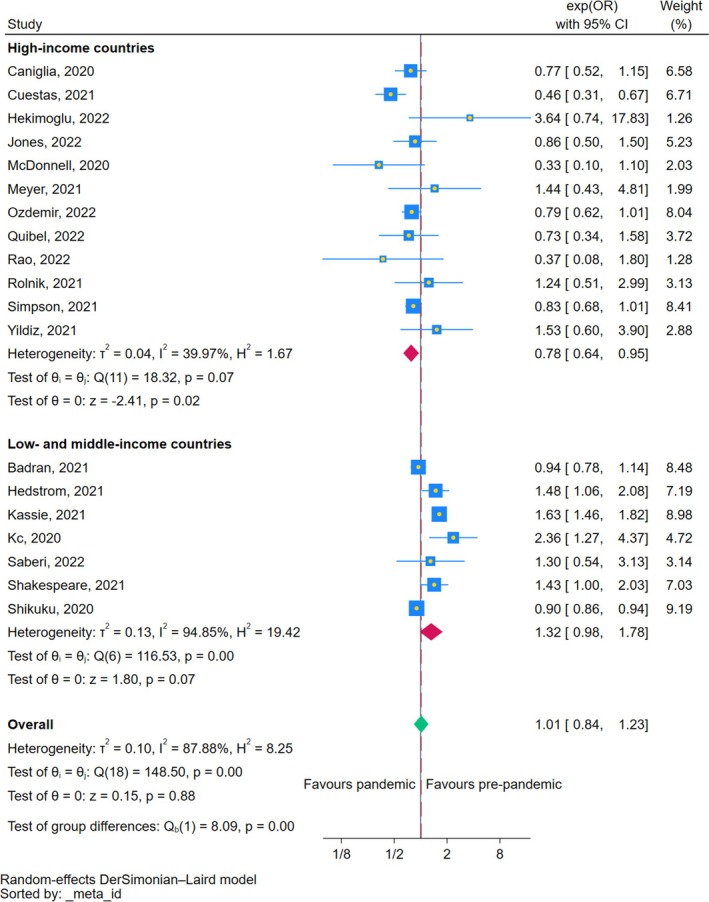
Rates of neonatal death in pre‐pandemic and pandemic periods according to World Bank Classification.

There was no difference in neonatal death rates during the pandemic versus pre‐pandemic period (19 studies, *n* = 12 300 infants): OR 1.01 (95% CI 0.84–1.23), *I* ^2^ = 0.88 (Figure 4). However, there was a decrease in neonatal death rates in HIC: OR 0.78 (95% CI 0.64–0.95), *I* ^*2*^ = 0.4. No effect of stringency of mitigation measures was observed (*p* = 0.88) (Figure S8).

### 7.6 Mode of Delivery

There was an increase in caesarean section rates during the pandemic compared to the pre‐pandemic period: 57 studies, 60 observational periods (*n* = 4 375 710), OR 1.05 (95% CI 1.03–1.07), *I* ^*2*^ = 0.97 (Figure S9a). This difference was observed in both HIC (OR 1.04 [95% CI 1.02–1.06], *I* ^*2*^ = 0.97) and LMIC (OR 1.07 [95% CI 1.03–1.13], *I* ^*2*^ = 0.82). There was no difference in caesarean section rates based on the stringency of mitigation measures implemented (*p* = 0.46) (Figure S9b).

## 8 Discussion

### 8.1 Principal Findings

This is the largest systematic review and meta‐analysis to report on the effects of the COVID‐19 pandemic on PTB, neonatal death and stillbirth, including 100 studies from 41 countries and 2 875 562 infants/pregnancies.

Rates of PTB declined during the pandemic compared with the pre‐pandemic period, with the most pronounced effect observed for extremely PTB in HICs. There was a significant decrease in neonatal death rates in HIC during the pandemic period, likely reflecting less infants born extremely preterm, when mortality risk is highest. Reassuringly, in HICs the reduction in both PTB and neonatal death during the pandemic occurred without evidence of increased stillbirth rates. In contrast, there was minimal change in PTB rates but a concerning increase in stillbirth rates in LMIC, highlighting the importance of awareness of the social and health service context when implementing changes to maternity care or pandemic responses. The paucity of data from LMIC reflects the urgent need to improve the collection of robust perinatal data in these regions, where the majority of PTB and perinatal deaths occur [24].

### 8.2 Research and Clinical Implications

There has been a lack of improvement in global PTB rates in the 20 years preceding the pandemic [25]. Although the reduction in PTB noted in this study is modest (5%–8%), this is the largest reduction experienced globally in decades. At a population level, this reduction could herald a very meaningful impact on global PTB and neonatal mortality. This warrants further investigation to determine what aspects of changes in health care or society response to the pandemic influenced this reduction and what may be replicated in the post‐pandemic era to pursue ongoing reductions.

There is no common pathway leading to PTB, occurring either spontaneously or be medically indicated due to concerns for maternal or foetal health. Our study only found a reduction in spontaneous PTB. The aetiology of spontaneous PTB is multifactorial, but maternal infection and inflammation are a common cause [26]. There is evidence that activation of the innate immune system in response to infection plays an important role in spontaneous preterm labour [27], and that this is likely of more importance in the pathophysiology of extremely PTB compared to births at later preterm gestations [28]. Given the greatest effect on PTB rate was in reduced extremely PTB, it is possible that reduced exposure to infections during pregnancy, as a result of social distancing, lockdowns, and improved hygiene, all of which may lead to a reduction in the transmission of infectious diseases, may have played a role in the reduction in this cohort [29]. Some of these behavioural changes may have been easier to implement for pregnant women in HICs compared with LMICs. In lower‐resourced settings it is possible that challenges with housing, finances, employment, childcare and access to maternity care may have restricted the ability for pregnant women to reduce their exposure to infections and thus the reduction in these regions was less pronounced [12, 30].

In contrast, there was no difference in the rates of medically indicated PTB. A potential concern was that restricted access to in‐person maternity care during lockdown may have reduced the detection of preterm foetal growth restriction and preeclampsia, a major antecedent of stillbirth, and the opportunity to expedite delivery to prevent perinatal mortality [31]. Reassuringly, there was no increase in stillbirths in HICs, although we were unable to capture information about maternal morbidity. We did see an increase in caesarean sections, although we were unable to determine if this was due to an increase in incidence of foetal distress or other obstetric indicators for operative delivery.

There was an increase in stillbirth in LMICs. The reasons for this are unclear but may be due to healthcare systems being under‐resourced to deal with the pandemic, with potential for obstetric or midwifery staff redeployment to care for COVID‐19 patients, and pregnant women may have been reluctant to seek medical help when indicated. Worryingly, this effect on LMIC stillbirth rates may be an underestimate, as the outcomes of home births are not recorded [12, 24, 32]. It is vital that the cause of this higher rate of stillbirths in LMIC is further interrogated to ensure this trend does not recur.

The Oxford Stringency index was used as a surrogate measure of the degree of mitigation strategies (lockdowns) implemented in a region in response to the pandemic. We found no difference in PTB rates according to the stringency of mitigation measures implemented, which suggests that there may be other confounding factors contributing. The variation in implementation of lockdowns, wide variations in SARS COV2 infection rates and testing strategies, and retrospective observational studies included here makes it challenging to establish causality. Other social and behavioural changes that may have been affected by the pandemic may also have played a role in changes to PTB rates. Whilst the relationship between physical work and preterm labour is unclear [27], it is possible that during lockdowns there was more sedentary behaviour and fewer physical demands, which could have a positive effect on PTB rates. Lockdown measures also had an effect in reducing anthropogenic emissions and improving air quality, which may also reduce PTB [33, 34, 35, 36]. These possible associations remain speculative.

### 8.3 Strengths and Limitations

Previous findings regarding the indirect effects of mitigation measures and perinatal outcomes have been varied and conflicting [13, 14, 15, 16, 17, 18]. The strengths of our review include the global representativeness of studies and the largest number of studies included in a systematic review of this topic. Outcomes such as stillbirth remain relatively rare, and no individual study is likely to be powered to detect trends, reflecting the importance of large meta‐analyses to detect evolving but important trends.

There are limitations of this study. There was a relative paucity of LMICs represented (13/100 studies), limiting the generalisability of these findings. This lack of representation of LMICs is not surprising, and there are likely to be significant gaps in reporting of perinatal outcomes during the pandemic in these regions due to reduction of institutional births and staff available for data collection [25]. Initiatives that are aiming to harmonise collection of robust high‐quality longitudinal data from both LMIC and HIC are critical to better inform international efforts to understand the impacts and potential mechanisms for changes in perinatal outcomes during pandemics [37]. We only included studies that reported temporal changes, but there was a wide variation in study design and duration of pre‐pandemic, pandemic and post‐pandemic reporting periods. Most studies included at least the previous calendar year; however, in some studies, the pre‐pandemic period was limited to the several months immediately preceding the implementation of mitigation strategies. It is recognised that there is variation in both stillbirth and PTB rates with seasonality and across time; therefore, studies that limited the pre‐pandemic period may have failed to detect changes independent of underlying temporal patterns [11]. There were also wide variations in the baseline PTB rates and population heterogeneity. Different methods were used to determine gestational age, and sometimes these were not fully described. Not all studies included both PTB, stillbirth and neonatal death rates as outcomes, with many studies focussing primarily on PTB. This limits the ability to interrogate if the reduction in PTB was at the expense of perinatal deaths or not [38]. Few studies reported the gestational age at time of stillbirth, limiting the capacity to draw conclusions regarding aetiology and mechanisms. Finally, we did not include other indicators of perinatal outcome including maternal deaths that may have also been impacted by the pandemic.

### 8.4 Conclusions

In conclusion, there was a modest but significant reduction in PTB rates during the pandemic period compared with the pre‐pandemic period, the most pronounced change observed in HICs. This reduction may have occurred at the expense of increased stillbirth rates in LMIC where the greatest burden on preterm birth and stillbirths occurs. Further research is required to understand the aetiology of these changes to ensure responses to similar future pandemics prioritise safe access to maternity care and robust data collection. These hypothesis‐generating findings provide future research opportunities to develop new interventions addressing changes in environmental exposures and the prevention of infection during pregnancy to improve perinatal outcomes.

## Author Contributions

Whitehead CL, Manley BJ and Cheong JLY conceptualised and designed the study, reviewed and revised the final manuscript. Peart SR designed the data collection instruments, collected data, drafted the initial manuscript, reviewed and revised the final manuscript. Haj‐Yahya R designed the data collection instruments, collected data, reviewed, and revised the final manuscript. Nugent M collected data and reviewed the manuscript. Harbinson L collected data and reviewed the manuscript. Ganbold O collected data, conducted the statistical analyses, and reviewed the manuscript.

## Funding

The authors have nothing to report.

## Conflicts of Interest

The authors declare no conflicts of interest.

## Supporting information


**Figure S1:** Search strategy.
**Table S1:** Description of included studies.
**Table S2:** Quality assessment of included studies using the Newcastle‐Ottawa Scale (NOS).
**Figure S2:** Funnel plot with bias test for rates of preterm birth (< 27 weeks' gestational age) in the pre‐pandemic and pandemic periods.
**Figure S3:** Forest plot of preterm birth in the pre‐pandemic and pandemic periods in high‐income versus low‐ and middle‐income countries: (a) Extremely preterm birth < 28 weeks' gestation. (b) Very preterm birth 28–31 weeks' completed gestation. (c) Moderate/late preterm birth 32–36 weeks' completed gestation.
**Figure S4:** Forest plot of preterm birth in the pre‐pandemic and pandemic periods according to stringency of mitigation measures.
**Figure S5:** Forest plot of (a) spontaneous preterm birth in the pre‐pandemic and pandemic periods in high‐income versus low‐ and middle‐income countries; (b) spontaneous preterm birth in the pre‐pandemic and pandemic periods according to stringency of mitigation measures; and (c) medically indicated preterm birth in the pre‐pandemic and pandemic periods in high‐income versus low‐and‐middle‐income countries.
**Figure S6:** Forest plot of low birth weight in the pre‐pandemic and pandemic periods according to high‐income versus low‐middle‐income countries for (a) < 2500 g, (b) < 1500 g.
**Figure S7:** Forest plot of stillbirth in the pre‐pandemic and pandemic periods according to stringency of mitigation measures.
**Figure S8:** Forest plot of neonatal death in the pre‐pandemic and pandemic periods according to stringency of mitigation measures.
**Figure S9:** Forest plot of caesarean section in the pre‐pandemic and pandemic periods according to (a) high‐income versus low‐middle income countries, (b) stringency of mitigation measures.

## Data Availability

Data will be available on request to the corresponding author.
