# Supplementary material for: Preterm Birth and Perinatal Mortality During the COVID‐19 Pandemic Period: A Systematic Review and Meta‐Analysis
Source: J Paediatr Child Health. 2026 May 2;62(6):924–35. doi: 10.1111/jpc.70403 (PMC13254033; doi:10.1111/jpc.70403)
Supplement: Supplementary file 1 — Figure S1: Search strategy. Table S1: Description of included studies. Table S2: Quality assessment of included studies using the Newcastle‐Ottawa Scale (NOS). Figure S2: Funnel plot with bias test for rates of preterm birth (< 27 weeks' gestational age) in the pre‐pandemic and pandemic periods. Figure S3: Forest plot of preterm birth in the pre‐pandemic and pandemic periods in high‐income versus low‐ and middle‐income countries: (a) Extremely preterm birth < 28 weeks' gestation. (b) Very preterm birth 28–31 weeks' completed gestation. (c) Moderate/late preterm birth 32–36 weeks' completed gestation. Figure S4: Forest plot of preterm birth in the pre‐pandemic and pandemic periods according to stringency of mitigation measures. Figure S5: Forest plot of (a) spontaneous preterm birth in the pre‐pandemic and pandemic periods in high‐income versus low‐ and middle‐income countries; (b) spontaneous preterm birth in the pre‐pandemic and pandemic periods according to stringency of mitigation measures; and (c) medically indicated preterm birth in the pre‐pandemic and pandemic periods in high‐income versus low‐and‐middle‐income countries. Figure S6: Forest plot of low birth weight in the pre‐pandemic and pandemic periods according to high‐income versus low‐middle‐income countries for (a) < 2500 g, (b) < 1500 g. Figure S7: Forest plot of stillbirth in the pre‐pandemic and pandemic periods according to stringency of mitigation measures. Figure S8: Forest plot of neonatal death in the pre‐pandemic and pandemic periods according to stringency of mitigation measures. Figure S9: Forest plot of caesarean section in the pre‐pandemic and pandemic periods according to (a) high‐income versus low‐middle income countries, (b) stringency of mitigation measures. [file JPC-62-924-s001.docx]

**Supplementary Material**

**Supplementary Figure 1 Search strategy**

**Supplementary Table 1 Description of included studies**

**Supplementary Table 2 Quality assessment of included studies using the Newcastle-Ottawa Scale (NOS)**

**Supplementary Figure 2: Funnel plot with bias test for rates of preterm birth (<27 weeks’ gestational age) in the pre-pandemic and pandemic periods**

**Supplementary Figure 3:** Forest plot of preterm birth in the pre-pandemic and pandemic periods in high-income versus low- and middle-income countries : (a) Extremely preterm birth <28 weeks’ gestation. (b) Very preterm birth 28-31 weeks’ completed gestation. (c) Moderate/late preterm birth 32-36 weeks’ completed gestation

**Supplementary** **Figure 4**: Forest plot of preterm birth in the pre-pandemic and pandemic periods according to stringency of mitigation measures

**Supplementary** **Figure 5**: Forest plot of (a) spontaneous preterm birth in the pre-pandemic and pandemic periods in high-income versus low- and middle-income countries; (b) spontaneous preterm birth in the pre-pandemic and pandemic periods according to stringency of mitigation measures; and (c) medically indicated preterm birth in the pre-pandemic and pandemic periods in high-income versus low-and-middle-income countries

**Supplementary Figure 6**: Forest plot of low birth weight in the pre-pandemic and pandemic periods according to high-income versus low-middle-income countries for (a) <2500g, (b) <1500g

**Supplementary Figure 7**: Forest plot of stillbirth in the pre-pandemic and pandemic periods according to stringency of mitigation measures

**Supplementary Figure 8**: Forest plot of neonatal death in the pre-pandemic and pandemic periods according to stringency of mitigation measures

**Supplementary Figure 9**: Forest plot of caesarean section in the pre-pandemic and pandemic periods according to (a) high-income versus low-middle income countries, (b) stringency of mitigation measures

**Supplementary Figure 1 Search strategy**

Search Strategy (Medline)

fetal mortality/ or perinatal mortality/ or Stillbirth/ or Sudden Infant Death/

00:01

2

Premature Birth/ or Obstetric Labor, Premature/

00:01

3

(stillborn or stillbirth or sudden infant death? or sids or cot death? or crib death? or ((neonat* or newborn or infan* or f?etal) adj4 (death* or mortality))).mp.

00:01

4

((preterm or premature*) adj3 (birth* or labo?r or deliver*)).mp.

00:08

5

((obstetric or neonat*) adj3 outcome*).mp.

00:05

6

1 or 2 or 3 or 4 or 5

00:02

7

exp Coronavirus/ or exp Coronavirus Infections/

00:01

8

(coronavirus* or corona virus* or ncov* or covid* or sars-cov* or sarscov* or Sars-coronavirus*).mp.

9

(7 or 8) not ((animals/ not humans/) or (bovine or calves or feline or porcine or canine or zoonotic or avian or murine or mouse or mice or rat or rats).tw.)

10

9 and (20191* or 202*).dp.

11

6 and 10

Search Strategy (Embase)

1

perinatal mortality/ or newborn mortality/ or fetus mortality/ or prenatal mortality/ or stillbirth/

00:01

2

premature labor/ or prematurity/ or immaturity/

00:01

3

(stillborn or stillbirth or sudden infant death? or sids or cot death? or crib death? or ((neonat* or newborn or infan* or f?etal) adj4 (death* or mortality))).mp.

00:01

4

((preterm or premature*) adj3 (birth* or labo?r or deliver*)).mp.

5

((obstetric or neonat*) adj3 outcome*).mp.

6

1 or 2 or 3 or 4 or 5

7

coronavirus disease 2019/ or coronavirinae/ or Coronavirus infection/

8

(coronavirus* or corona virus* or ncov* or covid* or sars-cov* or sarscov* or Sars-coronavirus*).mp.

9

7 or 8

10

6 and 9

11

10 and (20191* or 202*).dc. and human/

**Supplementary Table 1 Characteristics of studies included in the meta-analysis**

| Study | Country | Total No. | Number of participants – pre-pandemic | Number of participants – during pandemic | Centers involved | Pre pandemic period | Pandemic period | Stringency of lockdown | Inclusion criteria | Exclusion criteria | Outcomes reported | Newcastle-Ottawa Scale (NOS) |
| --- | --- | --- | --- | --- | --- | --- | --- | --- | --- | --- | --- | --- |
| Abdul-Mumin, 2021 | Ghana | 2901 | 1,616 | 1,285 | Single center | 1^st^ March 2019 – 31^st^ Aug 2019 | 1^st^ March 2020 – 31^st^ Aug 2020 | 86.1 | Admitted to unit and age up to 28 days | _ | PTB, LBW, mode of delivery | 8 |
| Adams, 2022 | Switzerland | 519,607 | 434,014 | 85,593 | National | 17^th^ March 2015 – 10^th^ May 2019 | 17^th^ March 2020 – 10^th^ May 2020 | 73.5 | All live births | _ | PTB | 9 |
| Alshaikh, 2021 | Canada | 28,517 | 24,160 | 4,357 | Regional | 16^th^ March 2015 – 15^th^ June 2019 | 16^th^ March 2020 – 15^th^ June 2020 | 74.5 | All preterm births 22-36+6 weeks' gestation | _ | PTB, Stillbirth, LBW | 9 |
| Arnaez, 2021 | Spain | 10,217 | 8,710 | 1,507 | Regional | 15 March - 3rd May 2015-2019 | 15th March - 3rd May 2020 | 85.2 | _ | _ | PTB, Stillbirth, LBW | 8 |
| Badran, 2021 | Jordan | 31,106 | 15,311 | 15,795 | National | 1^st^ May 2019 – 31^st^ March 2020 | 1^st^ April 2020 – 31^st^ Dec 2020 | 100 | All births between 24-42 weeks' | _ | PTB, Stillbirth, NND, LBW, mode of delivery | 9 |
| Bajaj, 2022 | USA | 328,879 | 250,896 | 77,983 | _ | 1^st^ March 2017 – 30^th^ Nov 2019 | 1^st^ March 2020 – 30^th^ Nov 2020 | 72.7 | All live births and fetal deaths | _ | PTB, Stillbirth, Mode of delivery | 9 |
| Berghella, 2020 | USA | 2108 | 911 | 1,197 | Single center | March 1st - July 31st 2019 | March 1st - July 31st 2020 | 72.7 | All births ≥20 weeks’ gestation |  | PTB, Perinatal Death | 8 |
| Bian, 2021 | China | 164,107 | 139,795 | 24,312 | Single center | 23^rd^ Jan 2014 – 30^th^ 2019 | 23^rd^ Jan 2020 – 30^th^ 2020 | 81.9 | All singleton pregnancies | Multiple births and infants delivered <24 weeks' gestation | PTB, Stillbirth, Mode of delivery | 9 |
| Briozzo, 2021 | Urguay | 6261 | 3,325 | 3,036 | Single center | March 15th - September 30th 2019 | March 15th - September 30th 2020 | 72.2 | _ | _ | PTB, LBW | 8 |
| Caniglia, 2020 | Botswana | 11905 | 8,316 | 3,589 | Multicenter (regional) | 3rd April - 7th May 2017-2019 | 3rd April - 7th May 2020 | 86.1 | Singletons >23+6 weeks | _ | PTB, Stillbirth, NND, LBW | 9 |
| Cesano, 2021 | Italy | 2475 | 1,215 | 1,260 | Single center | 1^st^ March 2019 – 30^th^ April 2020 | 1^st^ March 2020 – 30^th^ April 2020 | 93.5 | All pregnant women | _ | Stillbirth, Mode of delivery | 7 |
| Chen, 2022 | USA | 165,253 | 88,060 | 77,193 | National | 1^st^ April – 30^th^ June 2019 | 1^st^ April – 30^th^ June 2020 | 72.7 | singleton hospital deliveries at ≥20 weeks' gestation | _ | PTB, Stillbirth, | 7 |
| Cheung, 2021 | Canada, China | Canada: 11,665;  China:  25,503 | Canada: 6054; China: 13,776 | Canada: 5598; China: 11,727 | Multicenter (International) | Canada : 1st March - 30th April 2019 ; China 1st February -29th February 2019 | Canada : 1st March - 30th April 2020 ; China 1st February -29th February 2020 | Canada: 72.7; China 82 | _ | _ | PTB, Stillbirth | 7 |
| Cuestas, 2021 | Argentina | 16,555 | 8,437 | 8,118 | Regional | 13^th^ Aug – 31^st^ Dec 2019 | 13^th^ Aug – 31^st^ Dec 2020 | 92.6 | All pregnancies | _ | PTB, Stillbirth, NND, Mode of delivery | 9 |
| De Curtis, 2020 | Italy | 16,808 | 9,053 | 7,755 | Multicenter (regional) | 1^st^ March – 31^st^ May 2019 | March - April 2020; | 93.5 | Singletons | Multiple births; abortions for late termination for fetal abnormalities | PTB, Stillbirth, Mode of delivery | 6 |
| Dehaene, 2022 | Belgium | 10,589 | 5,307 | 5,282 | Regional | 1^st^ Jan – 31^st^ Dec 2019 | 1^st^ Jan – 31^st^ Dec 2020 | 81.5 | _ | _ | PTB | 7 |
| Dong, 2021 | China | 595,396 | 493,496 | 101,900 | Regional | 23^rd^ Jan 2015 – 24^th^ Feb 2019 | 23^rd^ Jan – 24^th^ Feb 2020 | 81.9 | Singleton live births | Multiple births, stillbirths or missing data | PTB, Stillbirth, Mode of delivery | 9 |
| Du, 2021 | China | 7694 | 4,509 | 3,185 | Single center | 20th May - 30th Nov 2019 | 20th Jan - 30th July 2020 | 82.0 | Singleton pregnancies | Multiple pregnancy | PTB, Stillbirth, LBW, Mode of delivery | 9 |
| Einarsdottir, 2021 | Iceland | 5021 | 3,963 | 1,058 | Single center | 1^st^ March 2016 – 31^st^ May 2019 | 1^st^ March – 31^st^ May 2020 | 53.7 | _ | _ | PTB, Mode of delivery | 9 |
| Ezenwa, 2021 | Nigeria | 375 | 254 | 121 | Single center | 1^st^ April – 30^th^ June 2019 | 1^st^ April – 30^th^ June 2020 | 85.7 | All deliveries | _ | PTB | 8 |
| Fisher, 2022 | USA | 16,544 | 8,246 | 8,298 | Single center | 1^st^ April – 30^th^ Nov 2019 | 1^st^ April – 30^th^ Nov 2020 | 72.7 | All births >20 weeks' gestation | _ | PTB | 9 |
| Gallo, 2022 | Australia | 25,696 | 23,627 | 2,069 | Single center | 16^th^ March 2013 – 17^th^ April 2019 | Early: 16^th^ March – 1^st^ May 2020 | 73.2 | Singleton pregnancies | _ | PTB, Stillbirth, Mode of delivery | 9 |
| Garabedian, 2021 | France | 7,922 | 4,093 | 3,829 | National | 22^nd^ Jan – 16^th^ March 20202 | 17^th^ March – 10^th^ May 2020 | 88.0 | Singleton births | _ | PTB, Stillbirth, Mode of delivery | 8 |
| Gurol-Urganci, 2022 | UK | 687,731 | 360,175 | 327,556 | National | 1^st^: 23^rd^ March – 24^th^ June 2019  2^nd^: 22^nd^ Sep 2019 – 23^rd^ Feb 2020 | 1^st^: 23^rd^ March – 24^th^ June 2020  2^nd^: 22^nd^ Sep 2020 – 23^rd^ Feb 2021 | 88.0 | Singleton births | _ | PTB, Stillbirth, Mode of delivery | 9 |
| Handley, 2021 | USA | 8,914 | 5,907 | 3,007 | Multicenter (regional) | 1^st^ March 2018 – 30th June 2019 | 1^st^ March – 30^th^ June 2020 | 72.7 | Singletons | _ | PTB, Stillbirth | 9 |
| Harvey, 2021 | USA | 49,845 | 41,713 | 8,132 | Regional | 22nd March 2015 - 30th April 2019 | 22nd March - 30th April 2020 | 72.2 | _ | Gestation <17 weeks | PTB, LBW, Mode of delivery | 9 |
| Hedermann, 2020 | Denmark | 31,180 | 26,018 | 5,162 | Multicenter (National) | 12^th^ March 2015- 14^th^ April 14th 2019 | 12^th^ March – 14^th^ April 2020 | 79.6 | Singletons >21+6 weeks | _ | PTB | 7 |
| Hedstrom, 2021 | Uganda | 1,186 | 619 | 567 | Single center | 1^st^ Oct 2019 – 31^st^ March 2020 | 1^st^ April – 30^th^ Sep 2020 | 93.5 | Patients admitted to the neonatal unit | _ | PTB, NND, LBW, mode of delivery | 5 |
| Hekimoglu, 2022 | Turkey | 307 | 154 | 153 | Single center | 1^st^ March – 20^th^ May 2019 | 1^st^ March – 30^th^ May 2020 | 77.8 | All babies admitted to the intensive care unit | _ | PTB, NND, Mode of delivery | 8 |
| Herzberger, 2022 | Israel | 5,369 | 2,701 | 2,668 | Single center | 1^st^ March – 23^rd^ July 2019 | 1^st^ March – 23^rd^ July 2020 | 91.7 | All women admitted to the delivery room | _ | PTB, Mode of delivery | 8 |
| Hui, 2022 | Australia | 74,624 | 49,892 | 24,732 | Regional | 6^th^ Nov 2017 – 3^rd^ June 2019 | 4^th^ Nov 2019 – 1^st^ June 2020 | 73.2 | Singleton pregnancies ≥24 weeks' gestation | Congenital anomalies, terminations of pregnancy, multiple gestations and non-Victorian residents. | PTB, Stillbirth | 9 |
| Huseynova, 2021 | Saudi Arabia | 7,226 | 5,463 | 1,763 | Single center | 1st March 2017 - 30th June 2019 | 1st March - 30th June 2020 | 94.5 | All live infants | Abortions, Stillbirths, IUFD | PTB | 7 |
| Hwang S, 2022 | USA | 291,365 | 245,106 | 46,259 | Regional | 1^st^ April 2015 – 31^st^ Dec 2019 | 1^st^ April – 31^st^ Dec 2020 | 72.7 | _ | <20 weeks' or >44 weeks', BW <300g | PTB, LBW, Mode of delivery | 9 |
| Hwang J, 2022 | South Korea | 3,736,447 | 3,481,423 | 255,024 | National | 1^st^ Jan 2011 – 31^st^ Dec 2019 | 1^st^ Jan – 31^st^ Dec 2020 | 82.4 | Singleton births | _ | PTB, LBW | 9 |
| Janevic, 2021 | USA | 8,026 | 4,192 | 3,834 | Regional | 28th March - 31st July 2019 | 28th March - 31st July 2020 | 72.7 | _ | _ | PTB | 9 |
| Jasper, 2022 | Australia | 22,941 | 22,034 | 907 | Single center | 1^st^ April 2018 – 31^st^ May 2019 | 1^st^ April – 31^st^ May 2020 | 73.2 | All births | _ | PTB, Stillbirth, Mode of delivery | 8 |
| Jeyamurugan, 2022 | USA | 787 | 427 | 360 | Single center | 1^st^ June – 31^st^ Dec 2019 | 1^st^ June – 31^st^ Dec 2020 | 72.7 | All preterm births 22-36+6 weeks' gestation | _ | PTB, Stillbirth, LBW, Mode of delivery | 7 |
| Jones, 2022 | UK | 159,357 | 130,326 | 29,031 | National | 1^st^ Jan 2016 – 31^st^ Dec 2019 | 1^st^ Jan – 31^st^ Dec 2020 | 88.0 | All births | _ | PTB, Stillbirth, NND, LBW, Mode of delivery | 9 |
| Justman, 2020 | Israel | 1,352 | 742 | 610 | Single center | 1^st^ March – 30^th^ April 2019 | 1^st^ March – 30^th^ April 2020 | 94.4 | All births | _ | PTB, Stillbirth, Mode of delivery | 8 |
| Kassie, 2021 | Ethiopia | 5,711 | 3,235 | 2,476 | Regional | 1^st^ March – 30^th^ June 2019 | 1^st^ March – 30^th^ June 2020 | 80.6 | All women who received reproductive and maternal healthcare services | _ | Stillbirth, NND Mode of delivery | 8 |
| Kc, 2020 | Nepal | 20,354 | 13,189 | 7,165 | National | 1st Jan – 31^st^ May 2019 | 1^st^ Jan – 31^st^ May 2020 | 96.3 | Singletons >21+6 weeks | Multiple births or >41+6. Weeks | PTB, Stillbirth, NND, Mode of delivery | 9 |
| Khalil, 2020 | United Kingdom | 3,399 | 1,681 | 1,718 | Single center | 1st Oct 2019 - 31st Jan 2020 | 1st Feb - 14th June 2020 | 79.6 | _ | Late terminations for fetal anomalies | PTB, Stillbirth, Mode of delivery | 7 |
| Kim, 2021 | South Korea | 3,011 | 2,765 | 246 | Single center | 22nd March 2011 - 31st October 2019 | 22nd March - 31st October 2020 | 82.4 | _ | Multiple pregnancy | PTB, Stillbirth, LBW, Mode of delivery | 8 |
| Kirchengast, 2021 | Austria | 946 | 277 | 669 | Single center | 1^st^ Jan – 29^th^ Feb 2020 | 1^st^ March – 31^st^ July 2020 | 81.5 | Singleton livebirths | COVID-19 Infected Mother | PTB, LBW, Mode of delivery | 7 |
| Klumper, 2021 | Netherlands | 134,250 | 107,326 | 26,924 | National | 15^th^ March 2015 – 15^th^ March 2018 | 15^th^ March – 15^th^ May 2020 | 78.7 | <32 weeks’ gestation | _ | PTB | 8 |
| Kumar, 2020 | India | 9,771 | 6,161 | 3,610 | Single center | 1^st^ March – 30^th^ September 2019 | 1^st^ March – 30^th^ September 2020 | 100 | _ | _ | Stillbirth | 9 |
| Leibovitch, 2021 | Israel | 103,542 | 77,903 | 25,639 | National | 11^th^ March 2017 – 5^th^ May 2019 | 11^th^ March – 5^th^ May 2020 | 94.4 | Live singleton births | Gestations < 22 weeks and > 44 weeks, multiple births | PTB | 8 |
| Lemon, 2021 | USA | 23,083 | 17,687 | 5,396 | Single center | 1^st^ Jan 2018 – 31^st^ Jan 2020 | 1^st^ April – 22^nd^ Oct 2020 | 72.7 | Singleton pregnancies | _ | PTB, Mode of delivery | 8 |
| Li, 2020 | China | 10,591 | 7,159 | 3,432 | Single center | 1st Jan 2019 - 22nd Jan 2020 | 23rd Jan - 14th March 2020 | 81.9 | Age 18-50 years old | Gestation <28 weeks, intrauterine fetal death | PTB, Mode of delivery | 9 |
| Lin, 2021 | China | 18,085 | 9,815 | 8,270 | Single center | 24^th^ Jan – 24^th^ March 2019 | 24^th^ Jan – 24^th^ March 2020 | 81.9 | Births >20 weeks’ gestation, received perinatal healthcare during lockdown period | COVID 19 infection, IVF pregnancy, twin or multiple births, not of Han race | PTB, Stillbirth, LBW | 9 |
| Lisonkova, 2022 | USA | 22,793,639 | 19,190,445 | 3,603,194 | National | 1^st^ Jan 2015 – 31^st^ Dec 2019 | 1^st^ Jan – 31^st^ Dec 2020 | 72.7 | Singleton or multiple births between 20 - 45 weeks’ gestation | Missing information on assisted reproductive therapy conception | Stillbirth | 7 |
| Litman, 2022 | USA | 579,976 | 271,444 | 308,532 | National | 1^st^ Jan 2019 - 28^th^ Feb 2020 | 1^st^ March 2020 – 31^st^ May 2021 | 72.7 | All hospital deliveries | _ | PTB, Stillbirth, Mode of delivery | 8 |
| Liu, 2021 | Canada | 1,266,054 | 1,062,481 | 203,573 | National | 1^st^ March 2015 – 31^st^ Aug 2019 | 1^st^ March – 31^st^ Aug 2020 | 76.4 | Singleton hospital deliveries >20 weeks’ gestation and spontaneous stillbirth | Deliveries in Quebec, late pregnancy terminations | PTB, Stillbirth | 8 |
| Maharajan, 2021 | India | 3,015 | 1,879 | 1,136 | Single center | 1^st^ Oct 2019 – 31^st^ March 2020 | 1^st^ April 2020 – 7^th^ July 2021 | 100 | _ | _ | PTB, Mode of delivery | * |
| Main, 2021 | USA | 713,567 | 580,714 | 132,853 | Regional | 1^st^ April 2016 – 31^st^ July 2019 | 1^st^ April – 31^st^ July 2020 | 72.2 | Singletons | _ | PTB | 9 |
| Maslin, 2022 | UK | 134,047 | 91,121 | 42,926 | Regional | 1^st^ Jan 2018 – 31^st^ Dec 2019 | 1^st^ Jan – 31^st^ Dec 2020 | 88.0 | Live births and neonatal admissions | Non-viable births with gestation >22 weeks and late terminations for fetal abnormalities | PTB | 8 |
| Matheson, 2021 | Australia | 4,962 | 2,514 | 2,448 | Single center | 1^st^ July – 30^th^ September 2019 | 1^st^ July – 30^th^ September 2020 | 73.2 | All hospital deliveries | Pregnancy terminations | PTB, Stillbirth, LBW | 8 |
| McDonnell, 2020 | Ireland | 11,224 | 8,089 | 3,135 | Single center | 1^st^: 1^st^ March 2018 – 31^st^ July 2019  2^nd^: 1^st^ Jan – 29^th^ Feb 2020 | 1^st^: 1^st^ March – 31^st^ July 2020 | 90.7 | _ | _ | PTB, Stillbirth, NND, LBW Mode of delivery | 8 |
| Melov, 2022 | Australia | 34,103 | 23,722 | 10,381 | Regional | 1^st^ Jan 2018 – 31^st^ Jan 2020 | 1st Feb 2020 – 31^st^ Jan 2021 | 73.2 | Singleton pregnancies > 20 weeks’ gestation | Pregnancy terminations | PTB, Stillbirth, Mode of delivery | 9 |
| Meyer, 2021 | Israel | 31,280 | 28,686 | 2,594 | Single center | 20th March 2011 - 27th June 2019 | 20th March - 27th June 2020 | 94.4 | Singletons | _ | PTB, Stillbirth, NND, Mode of delivery | 7 |
| Meyer, 2022 | Israel | 23,531 | 10,707 | 12,824 | Single center | 19^th^ March 2019 – 19^th^ March 2020 | 20^th^ March 2020 – 3^rd^ June 2021 | 94.4 | All deliveries >24 weeks’ gestation, first newborn if multiple gestations | Pregnancy terminations | PTB, Stillbirth, Mode of delivery | 8 |
| Mikus, 2021 | Croatia | 6,009 | 3,277 | 2,732 | Single center | 25^th^ Feb – 31^st^ Dec 2019 | 25^th^ Feb – 31^st^ Dec 2020 | 96.3 | Singleton pregnancies | _ | PTB, Stillbirth, Mode of delivery | 8 |
| Molholm, 2022 | Denmark | 304,813 | 244,481 | 60,332 | National | 1^st^ March 2016 – 28^th^ Feb 2020 | 1^st^ March 2020 – 28^th^ Feb 2021 | 72.2 | Births from 22+0 to 36+6 weeks gestation | Pregnancy terminations | PTB, Stillbirth | 8 |
| Molina, 2022 | USA | 1,654,868 | 849,544 | 805,324 | Regional | 1^st^ Jan 2019 – 28^th^ Feb 2020 | 1^st^ March 2020 – 30^th^ April 2021 | 72.7 | Preterm births 24 - 36 weeks’ gestation | _ | PTB | 8 |
| Molina-Merino, 2021 | Spain | 60,962 | 51,484 | 9,478 | Regional | 14^th^ March 2015 – 21^st^ June 2019 | 14^th^ March – 21^st^ June 2020 | 85.2 | Preterm births 24 - 36 weeks’ gestation | _ | PTB | 8 |
| Mor, 2020 | Israel | 6,120 | 4,564 | 1,556 | Single center | 21st February 2017 - 30th April 2019 | 21st February - 30th April 2020 | 94.4 | Singletons | Gestation <24 weeks; multiple births; births following feticide | PTB, Stillbirth, Mode of delivery | 7 |
| Muin, 2021 | Austria | 411,986 | 346,497 | 65,489 | National | 1^st^ March 2015 – 31^st^ Dec 2019 | 1^st^ March – 31^st^ Dec 2020 | 81.5 | All live births and stillborn deliveries at or after 24 + 0 gestation | Multiple pregnancies, intrapartum and perinatal deaths, and stillborn fetuses following late ter- minations of pregnancy and those with congenital anomalies | PTB, Stillbirth | 8 |
| Mullin, 2022 | USA | 10,610 | 7,163 | 3,447 | Single center | 10^th^ March 2018 – 31^st^ Dec 2019 | 10^th^ March – 31^st^ Dec 2020 | 72.7 | Pregnancies >20 weeks’ gestation + singleton births | COVID 19 infection of the mother | PTB, Stillbirth | 9 |
| Naqvi, 2022 | Kenya, Zambia, the Democratic Republic of the Congo, Pakistan, India & Guatemala | 57,783 | 30,236 | 27,457 | International | 1^st^ March 2019 – 28^th^ Feb 2020 | 1^st^ March 2020 – 28^th^ Feb 2021 | 88.8 | Births >20 weeks’ gestation | _ | PTB, Stillbirth, NND, LBW, Mode of delivery, | 9 |
| Noddin, 2021 | USA | 304,023 | 152,832 | 151,191 | National | 1^st^ Oct 2019 – 31^st^ March 2020 | 1^st^ April – 30^th^ Sep 2020 | 72.7 | Singleton deliveries | _ | PTB, Mode of delivery | 7 |
| Oakley, 2022 | Norway, Sweden & Denmark | 1,519,521 | 1,306,564 | 212,957 | International | 1^st^ Jan 2014 – 31^st^ Dec 2019 | 1^st^ Jan – 31^st^ Dec 2020 | 69.4 - 79.6 | Births >22 weeks’ gestation | Second or higher order births from a multiple pregnancy | PTB | 8 |
| Ohashi, 2022 | Japan | 17,746 | 11,790 | 5,956 | National | 1^st^ Oct 2018 – 31^st^ Dec 2019 | 1^st^ Oct - 31^st^ Dec 2020 | 47.2 | All births | _ | PTB, LBW, Mode of delivery | 8 |
| Ornaghi, 2021 | Italy | 3,666 | 1,882 | 1,784 | Single center | 1^st^ March – 30^th^ Nov 2019 | 1^st^ March – 30^th^ Nov 2020 | 93.5 | Asymptomatic, COVID negative mothers who gave birth in the time period | COVID 19 infection of the mother | PTB, Stillbirth, Mode of delivery | 8 |
| Ozdemir, 2022 | Turkey | 41,628 | 28,021 | 13,607 | Regional | 1^st^ March 2018 – 29^th^ Feb 2020 | 1^st^ March 2020 – 28^th^ Feb 2021 | 77.8 | Stillbirths and infant deaths in the region | _ | Stillbirth, NND | 8 |
| Pasternak, 2021 | Sweden | 108,923 | 91,262 | 17,661 | National | 1st April 2015 - 31st May 2019 | 1st April - 31st May 2020 | 64.8 | Singleton births | Multiple births | PTB, Stillbirth | 8 |
| Philip, 2020 | Ireland | 7,092 | 5,768 | 1,324 | Single center | 1^st^ March 2016 – 30^th^ June 2019 | 1^st^ March – 30^th^ June 2020 | 90.7 | Births from 22 weeks’ gestation | _ | Stillbirth, LBW | 7 |
| Quibel, 2022 | France | 8,074 | 4,169 | 3,905 | National | 22^nd^ Jan – 16^th^ March 2020 | 17^th^ March – 10^th^ May 2020 | 88.0 | Singleton and multiple pregnancies birthed >24 weeks’ gestation and >500g | Termination of pregnancy, home birth | PTB, Stillbirth, NND, Mode of delivery | 8 |
| Ranjbar, 2021 | Iran | 2,503 | 1,287 | 1,216 | Single center | 19^th^ Feb – 19^th^ April 2019 | 19^th^ Feb – 19^th^ April 2020 | 72.6 | Singleton pregnancies | _ | PTB, Stillbirth, LBW, Mode of delivery | 8 |
| Rao, 2022 | USA | 2,442 | 1,384 | 1,058 | Single center | 27^th^ March – 31^st^ May 2019 | 27^th^ March – 31^st^ May 2020 | 72.7 | All singleton births | Mothers < 18 years, COVID positive, fetal anomaly or multiple gestations | PTB, NND, Mode of delivery | 9 |
| Riley, 2021 | USA | 99,422 | 78,718 | 20,704 | Regional | 1^st^ Jan 2017 – 29^th^ Feb 2020 | 1^st^ March – 31^st^ Dec 2020 | 72.7 | All singleton births 20 – 42+6 weeks’ gestation | _ | PTB, Mode of delivery | 8 |
| Roberts, 2021 | Canada | 799,893 | 705,767 | 94,126 | Regional | 1^st^ March 2015 – 29^th^ Feb 2020 | 1^st^ March – 31^st^ Oct 2020 | 76.4 | All pregnancies who delivered >20 weeks’ gestation | _ | PTB, Stillbirth, Mode of delivery | 8 |
| Rolnik, 2021 | Australia | 6,416 | 3,229 | 3,187 | Regional | 1^st^ Nov 2018 – 28^th^ Feb 2019 | 1^st^ Nov 2019 – 28^th^ Feb 2020 | 73.2 | Conception date or birth > 20weeks’ gestation in specified time period | Major fetal abnormality | PTB, Stillbirth, NND, LBW Mode of delivery | 9 |
| Saberi, 2022 | Iran | 20,382 | 10,485 | 9,897 | Regional | 21^st^ March – 21^st^ Dec 2019 | 20th March – 20th^t^ Dec 2020 | 72.6 | All pregnancies | _ | PTB, Stillbirth, NND, LBW Mode of delivery | 8 |
| Salerno, 2022 | Italy | 219,026 | 179,851 | 39,175 | Regional | 1^st^ Nov 2014 – 20^th^ Feb 2020 | 1^st^ March 2020 – 30^th^ June 2021 | 93.5 | Stillbirths | _ | Stillbirth | 8 |
| Shah, 2021 | Canada | 2,451,606 | 2,324,866 | 126,740 | Regional | 1^st^ July 2002 – 31^st^ Dec 2019 | 1^st^ Jan – 31^st^ Dec 2020 | 76.4 | All hospital deliveries | Abortions before 20 weeks GA, birth weight <500g | PTB, Stillbirth | 8 |
| Shakespeare, 2021 | Zimbabwe | 4,285 | 2,242 | 2,043 | Single center | 1^st^ Jan – 31^st^ March 2020 | 1^st^ April – 30^th^ June 2020 | 87.9 | All hospital deliveries with mothers aged 15 - 45 years | _ | Stillbirth, NND, Mode of delivery | 7 |
| Shikuku, 2020 | Kenya | 793,390 | 394,852 | 398,538 | National | 1^st^ March – 30^th^ June 2019 | 1^st^ March – 20^th^ June 2020 | 88.9 | _ | _ | Stillbirth, NND, Mode of delivery | 8 |
| Simeone, 2022 | USA | 1,277,713 | 663,620 | 614,093 | National | 1^st^ April – 31^st^ Dec 2019 | 1^st^ April – 31^st^ Dec 2020 | 72.7 | Singleton births or stillbirths >20 and <42 weeks’ gestation | _ | PTB, Stillbirth, Mode of delivery | 9 |
| Simpson, 2021 | Canada | 416,380 | 348,633 | 67,747 | Regional | 15th March 2015 - 30th September 2019 | 15th March - 30th September 2020 | 76.4 | In-hospital births at 20 weeks’ or more gestational age | _ | PTB, Stillbirth, NND, LBW | 9 |
| Son, 2021 | USA | 838,489 | 613,264 | 225,225 | National | 1^st^ March 2017 – 31^st^ Dec 2019 | 1^st^ March – 31^st^ Dec 2020 | 72.7 | Births > 4 weeks’ gestation born in hospitals using EPIC software with >100 annual births annually. | Multiple gestation, missing outcomes | PTB, Stillbirth | 9 |
| Stowe, 2020 | UK | 270,963 | 139,745 | 131,218 | National | 1^st^ April – 30^th^ June 2019 | 1^st^ April – 30^th^ June 2020 | 79.6 | Hospitalizations for stillbirths within the NHS | _ | Stillbirth | 8 |
| Stumpfe, 2022 | Germany | 361,737 | 326,404 | 35,333 | Regional | 1^st^: 16^th^ March 2010 – 5^th^ May 2019  2^nd^: 2^nd^ Nov 2010 – 31^st^ Dec 2019 | 1^st^: 16^th^ March – 5^th^ May 2020  2^nd^: 2^nd^ Nov – 31^st^ Dec 2020 | 82.4 | Singleton and twin births | Higher order multiple pregnancies | PTB, LBW | 9 |
| Vani, 2022 | USA | 12,863 | 9,878 | 2,985 | Single center | 1^st^ March 2017 – 30^th^ Sep 2019 | 1^st^ March – 30^th^ Sep 2020 | 72.7 | Stillbirths induced and delivered in the specified centre | Fetal death <20 weeks’ gestation, birth weight <500g, spontaneous pre-viable preterm delivery, inducted terminations, twin pregnancies | Stillbirth | 9 |
| Wang B, 2021 | China | 32,242 | 18,621 | 13,621 | Regional | 1^st^ Jan – 31^st^ May 2019 | 1^st^ Jan – 31^st^ May 2020 | 81.9 | All pregnant women with delivery or termination in the specified regions | COVID 19 infection of the mother | PTB, Stillbirth, LBW, Mode of delivery | 8 |
| Wang J, 2021 | China | 1,322 | 733 | 589 | Single center | 24^th^ Jan – 31^st^ March 2019 | 24^th^ Jan – 31^st^ March 2020 | 81.9 | Uncomplicated singleton pregnancies | Maternal characteristics of COVID 19 or other viral infection, diabetes, hypertension, thalassaemia, twin/multiple pregnancies, IVF | PTB, LBW, Mode of delivery | 8 |
| Wen, 2022 | China | 112,542 | 92,750 | 19,792 | Single center | 1^st^ Jan 2016 – 31^st^ Dec 2019 | 1^st^ Jan – 31^st^ Dec 2020 | 81.9 | All singleton births | Multiples; ≤28 weeks' gestation | PTB, LBW | 9 |
| Wood, 2021 | USA | 9,356 | 4,644 | 4,712 | Regional | 1^st^ April – 31^st^ July 2019 | 1^st^ April – 31^st^ July 2020 | 72.7 | Singleton livebirths >19+6 weeks | _ | PTB | 7 |
| Xie, 2022 | China | 11,714,947 | 10,693,951 | 1,020,996 | National | 1^st^ Jan 2012 – 31^st^ Jan 2020 | 1^st^ Jan – 31^st^ Dec 2020 | 81.9 | All deliveries >28 weeks' gestation or >1000g | _ | PTB, Stillbirth | 9 |
| Yalcin, 2022 | Turkey | 2,219914 | 1,141,385 | 1,078,529 | Regional | 1^st^ Jan – 31^st^ Dec 2019 | 1^st^ Jan – 31^st^ Dec 2020 | 77.8 | Hospital births | Birth weight <500g or <22 weeks' gestation | PTB, LBW, Mode of delivery | 9 |
| Yildiz, 2021 | Turkey | 1,805 | 1,304 | 501 | Regional | 11^th^ March 2018 – 1^st^ June 2019 | 11^th^ March – 1^st^ June 2020 | 77.8 | Pregnant women attending hospital visits | _ | Stillbirth, NND, Mode of delivery | 8 |

Abbreviations : IUFD - Intra-Uterine Fetal Death; NHS – National Health Service; PTB – Preterm Birth; LBW – Low Birth Weight; NND- neonatal Death; NICU – Neonatal Intensive Care Unit

**Supplementary Table 2 Quality assessment of included studies using the Newcastle-Ottawa Scale (NOS)**

| Author | Representativeness of exposed cohort (max score: *) | Selection of the non exposed cohort (max score: *) | Ascertainment of exposure (max score: *) | Demonstration that outcome of interest was not present at start of study (max score: *) | Comparability of cohorts on the basis of the design or analysis (max score: **) | Assessment of outcome (max score: *) | Was follow-up long enough for outcomes to occur (max score: *) | Adequacy of follow up of cohorts (max score: *) | Total (max score: 9*) |
| --- | --- | --- | --- | --- | --- | --- | --- | --- | --- |
| Abdul-Mumin, 2021 | * | * | * | * | *- | * | * | * | 8* |
| Adams, 2022 | * | * | * | * | ** | * | * | * | 9* |
| Alshaikh, 2021 | * | * | * | * | ** | * | * | * | 9* |
| Arnaez, 2021 | * | * | * | * | * | * | * | * | 8* |
| Badran, 2021 | * | * | * | * | ** | * | * | * | 9* |
| Bajaj, 2022 | * | * | * | * | ** | * | * | * | 9* |
| Berghella, 2020 | * | * | * | * | * | * | * | * | 8* |
| Bian, 2021 | * | * | * | * | ** | * | * | * | 9* |
| Briozzo, 2021 | * | * | * | * | * | * | * | * | 8* |
| Caniglia, 2020 | * | * | * | * | ** | * | * | * | 9* |
| Cesano, 2021 | * | * | * | * | -- | * | * | * | 7* |
| Chen, 2022 | - | * | * | * | *- | * | * | * | 7* |
| Cheung, 2021 | - | * | * | * | -- | * | * | * | 7* |
| Cuestas, 2021 | * | * | * | * | ** | * | * | * | 9* |
| De Curtis, 2020 | - | * | * | * | -- | * | * | * | 6* |
| Dehaene, 2022 | * | * | * | * | -- | * | * | * | 7* |
| Dong, 2021 | * | * | * | * | ** | * | * | * | 9* |
| Du, 2021 | * | * | * | * | ** | * | * | * | 9* |
| Einarsdottir, 2021 | * | * | * | * | ** | * | * | * | 9* |
| Ezenwa, 2021 | * | * | * | * | * | * | * | * | 8* |
| Fisher, 2022 | * | * | * | * | ** | * | * | * | 9* |
| Gallo, 2022 | * | * | * | * | ** | * | * | * | 9* |
| Garabedian, 2021 | * | * | * | * | * | * | * | * | 8* |
| Gurol-Urganci, 2022 | * | * | * | * | ** | * | * | * | 9* |
| Handley, 2021 | * | * | * | * | ** | * | * | * | 9* |
| Harvey, 2021 | * | * | * | * | ** | * | * | * | 9* |
| Hedermann, 2020 | * | * | * | * | -- | * | * | * | 7* |
| Hedstrom, 2021 | - | * | * | * | -- | - | * | * | 5* |
| Hekimoglu, 2022 | * | * | * | * | * | * | * | * | 8* |
| Herzberger, 2022 | * | * | * | * | * | * | * | * | 8* |
| Hui, 2022 | * | * | * | * | ** | * | * | * | 9* |
| Huseynova, 2021 | * | * | * | * | -- | * | * | * | 7* |
| Hwang S, 2022 | * | * | * | * | ** | * | * | * | 9* |
| Hwang J, 2022 | * | * | * | * | ** | * | * | * | 9* |
| Janevic, 2021 | * | * | * | * | ** | * | * | * | 9* |
| Jasper, 2022 | * | * | * | * | * | * | * | * | 8* |
| Jeyamurugan, 2022 | - | * | * | * | * | * | * | * | 7* |
| Jones, 2022 | * | * | * | * | ** | * | * | * | 9* |
| Justman, 2020 | * | * | * | * | * | * | * | * | 8* |
| Kassie, 2021 | * | * | * | * | * | * | * | * | 8* |
| Kc, 2020 | - | * | * | * | * | * | * | * | 7* |
| Khalil, 2020 | * | * | * | * | -- | * | * | * | 7* |
| Kim, 2021 | * | * | * | * | * | * | * | * | 8* |
| Kirchengast, 2021 | * | * | * | * | -- | * | * | * | 7* |
| Klumper, 2021 | * | * | * | * | * | * | * | * | 8* |
| Kumar, 2020 | * | * | * | * | ** | * | * | * | 9* |
| Leibovitch, 2021 | * | * | * | * | * | * | * | * | 8* |
| Lemon, 2021 | * | * | * | * | * | * | * | * | 8* |
| Li, 2020 | * | * | * | * | ** | * | * | * | 9* |
| Lin, 2021 | * | * | * | * | ** | * | * | * | 9* |
| Lisonkova, 2022 | - | * | * | * | * | * | * | * | 7* |
| Litman, 2022 | * | * | * | * | * | * | * | * | 8* |
| Liu, 2021 | * | * | * | * | * | * | * | * | 8* |
| Mahajan, 2021 | - | * | * | * | -- | * | * | * | 6* |
| Main, 2021 | * | * | * | * | ** | * | * | * | 9* |
| Maslin, 2022 | * | * | * | * | * | * | * | * | 8* |
| Matheson, 2021 | * | * | * | * | * | * | * | * | 8* |
| McDonnell, 2020 | * | * | * | * | * | * | * | * | 8* |
| Melov, 2022 | * | * | * | * | ** | * | * | * | 9* |
| Meyer, 2021 | * | * | * | * | -- | * | * | * | 7* |
| Meyer, 2022 | * | * | * | * | * | * | * | * | 8* |
| Mikus, 2021 | * | * | * | * | * | * | * | * | 8* |
| Molholm, 2022 | * | * | * | * | * | * | * | * | 8* |
| Molina, 2022 | * | * | * | * | * | * | * | * | 8* |
| Molina-Merino, 2021 | * | * | * | * | * | * | * | * | 8* |
| Mor, 2020 | * | * | * | * | -- | * | * | * | 7* |
| Muin, 2021 | * | * | * | * | * | * | * | * | 8* |
| Mullin, 2022 | * | * | * | * | ** | * | * | * | 9* |
| Naqvi, 2022 | * | * | * | * | ** | * | * | * | 9* |
| Noddin, 2021 | * | * | * | * | -- | * | * | * | 7* |
| Oakley, 2022 | * | * | * | * | * | * | * | * | 8* |
| Ohashi, 2022 | * | * | * | * | * | * | * | * | 8* |
| Ornaghi, 2021 | * | * | * | * | * | * | * | * | 8* |
| Ozdemir, 2022 | * | * | * | * | * | * | * | * | 8* |
| Pasternak, 2021 | * | * | * | * | * | * | * | * | 8* |
| Philip, 2020 | * | * | * | * | -- | * | * | * | 7* |
| Quibel, 2022 | * | * | * | * | * | * | * | * | 8* |
| Ranjbar, 2021 | * | * | * | * | * | * | * | * | 8* |
| Rao, 2022 | * | * | * | * | ** | * | * | * | 9* |
| Riley, 2021 | * | * | * | * | * | * | * | * | 8* |
| Roberts, 2021 | * | * | * | * | * | * | * | * | 8* |
| Rolnik, 2021 | * | * | * | * | ** | * | * | * | 9* |
| Saberi, 2022 | * | * | * | * | * | * | * | * | 8* |
| Salerno, 2022 | * | * | * | * | * | * | * | * | 8* |
| Shah, 2021 | * | * | * | * | * | * | * | * | 8* |
| Shakespeare, 2021 | * | * | * | * | -- | * | * | * | 7* |
| Shikuku, 2020 | * | * | * | * | * | * | * | * | 8* |
| Simeone, 2022 | * | * | * | * | ** | * | * | * | 9* |
| Simpson, 2021 | * | * | * | * | ** | * | * | * | 9* |
| Son, 2021 | * | * | * | * | ** | * | * | * | 9* |
| Stowe, 2020 | * | * | * | * | -- | * | * | * | 8* |
| Stumpfe, 2022 | * | * | * | * | ** | * | * | * | 9* |
| Vani, 2022 | * | * | * | * | ** | * | * | * | 9* |
| Wang B, 2021 | * | - | * | * | ** | * | * | * | 8* |
| Wang J, 2021 | * | * | * | * | ** | * | * | - | 8* |
| Wen 2022 | * | * | * | * | ** | * | * | * | 9* |
| Wood, 2021 | * | * | * | * | -- | * | * | * | 7* |
| Xie, 2022 | * | * | * | * | ** | * | * | * | 9* |
| Yalcin, 2022 | * | * | * | * | ** | * | * | * | 9* |
| Yildiz, 2022 | * | * | * | * | * | * | * | * | 8* |

**Supplementary Figure 2 Funnel plot with bias test for rates of preterm birth (<27 weeks’ gestational age) in the pre-pandemic and pandemic periods**

**
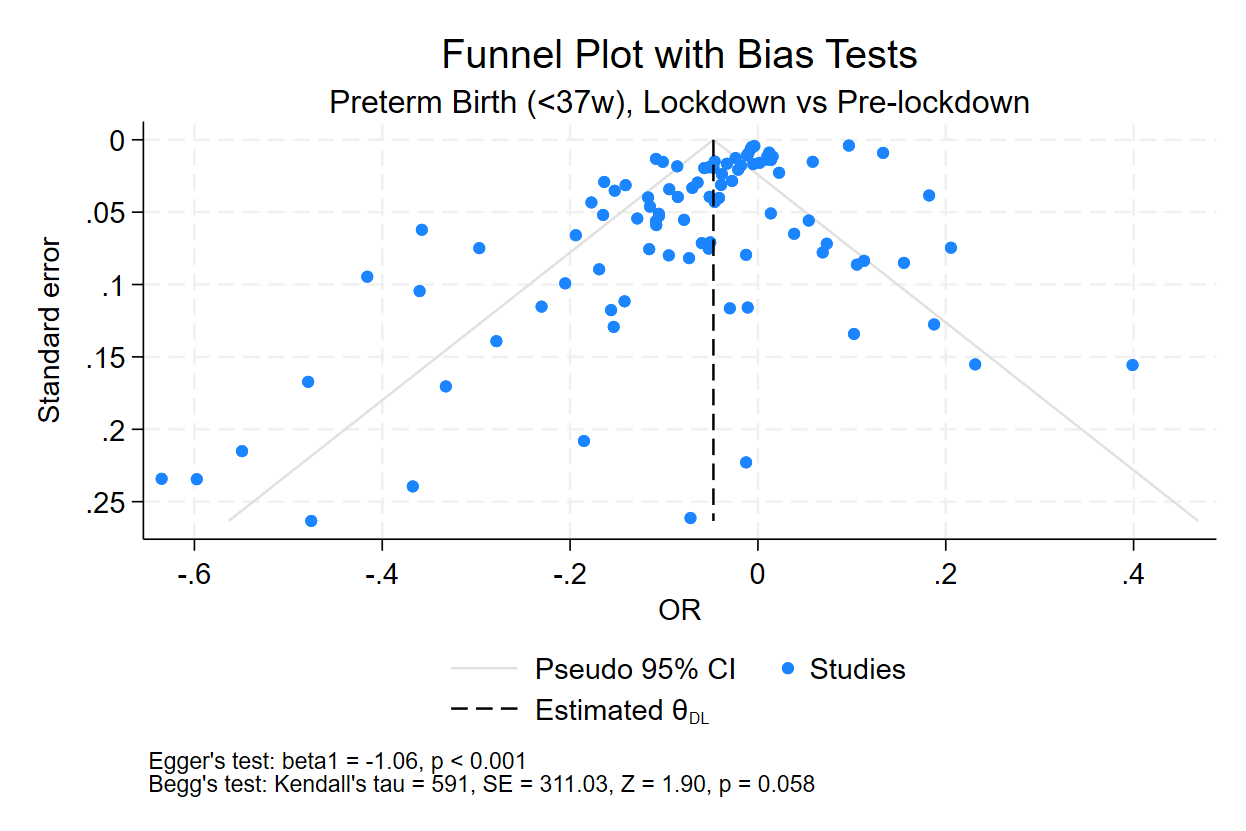
**

**Supplementary Figure 3a Rates of extremely preterm birth (<28 weeks’ gestational age) in the pre-pandemic and pandemic periods according to World Bank Classification**


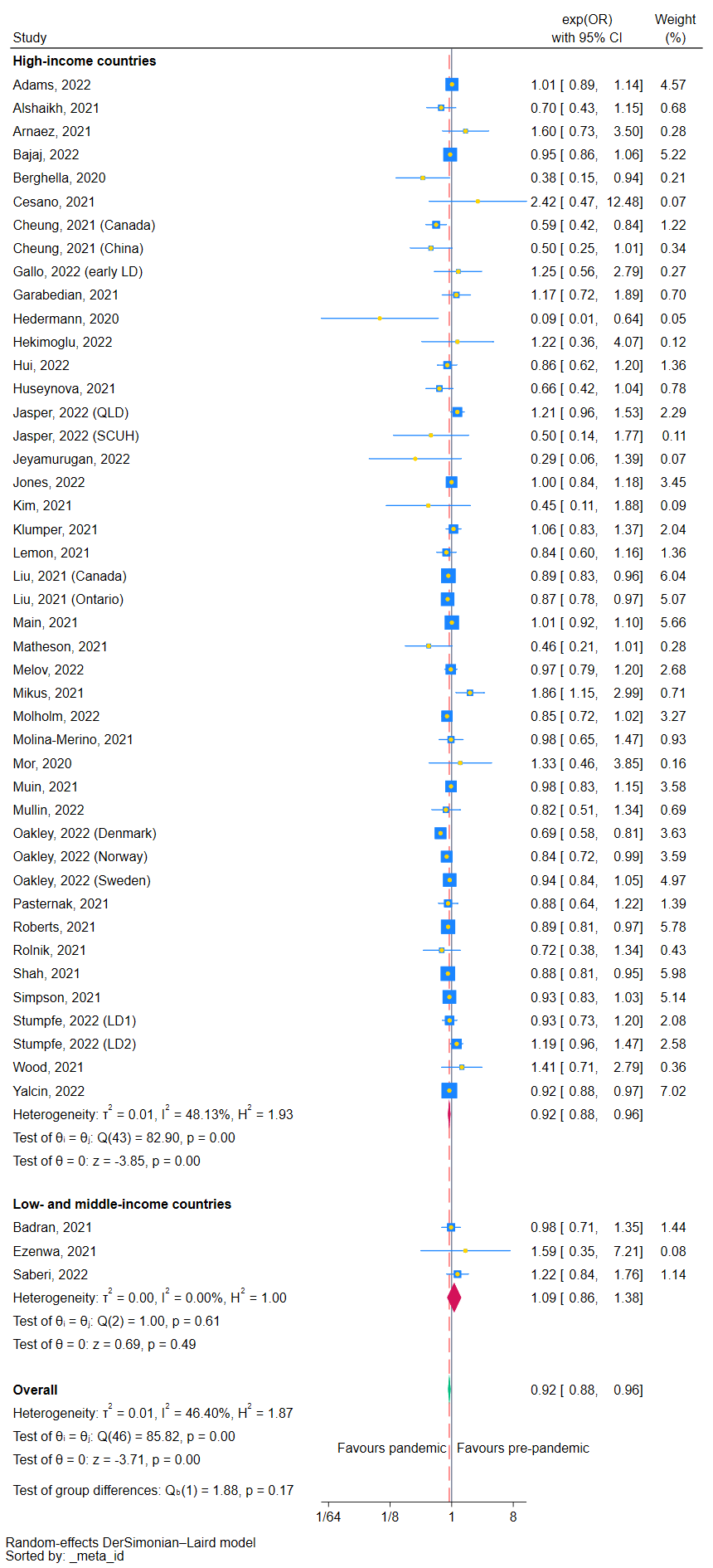


**Supplementary Figure 3b Rates of very preterm birth (28-31 weeks’ completed gestation) in the pre-pandemic and pandemic periods according to World Bank Classification**

**
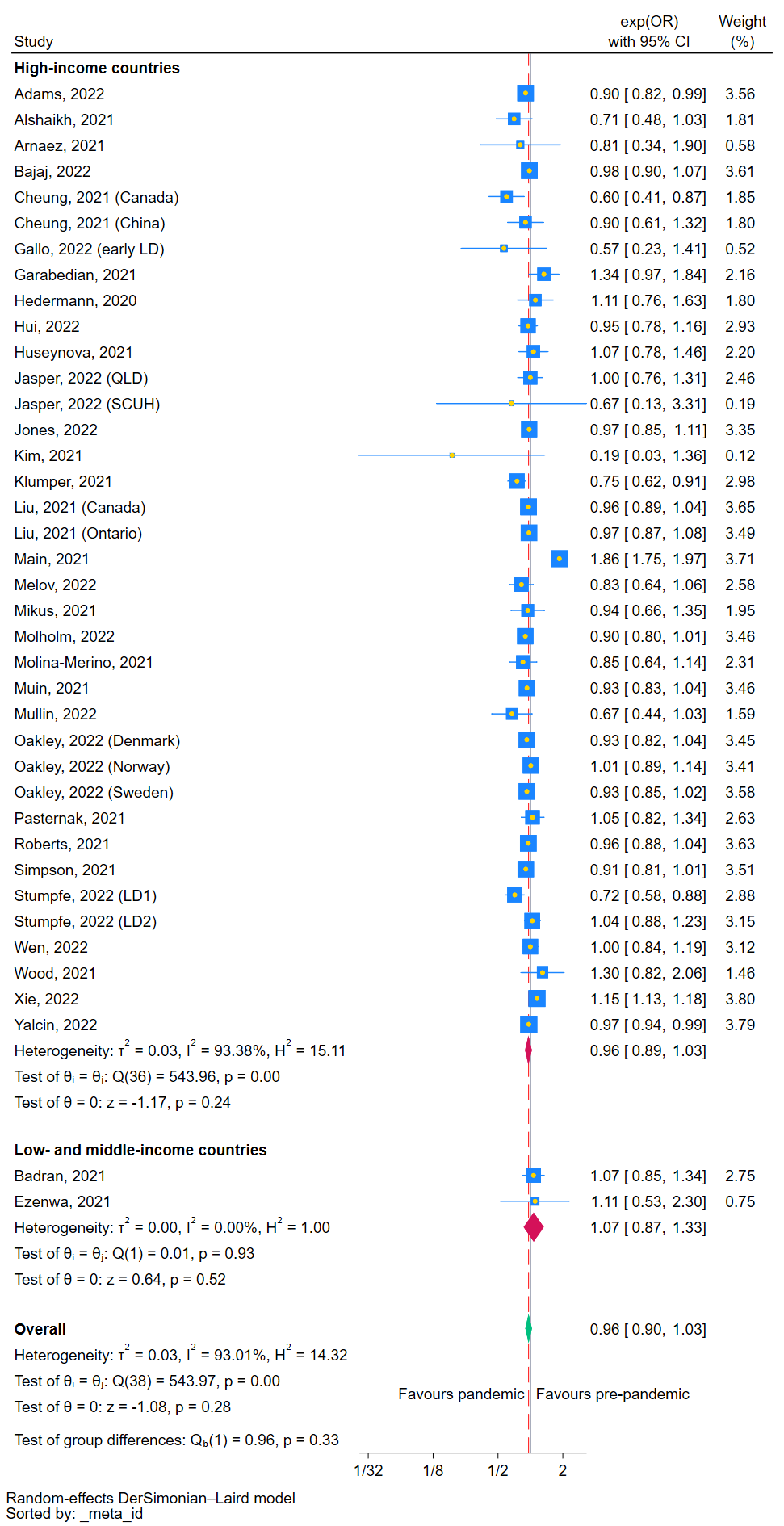
**

**Supplementary Figure 3c Rates of moderate-late preterm births (32-36 weeks’ completed gestation) in the pre-pandemic and pandemic periods according to World Bank Classification**


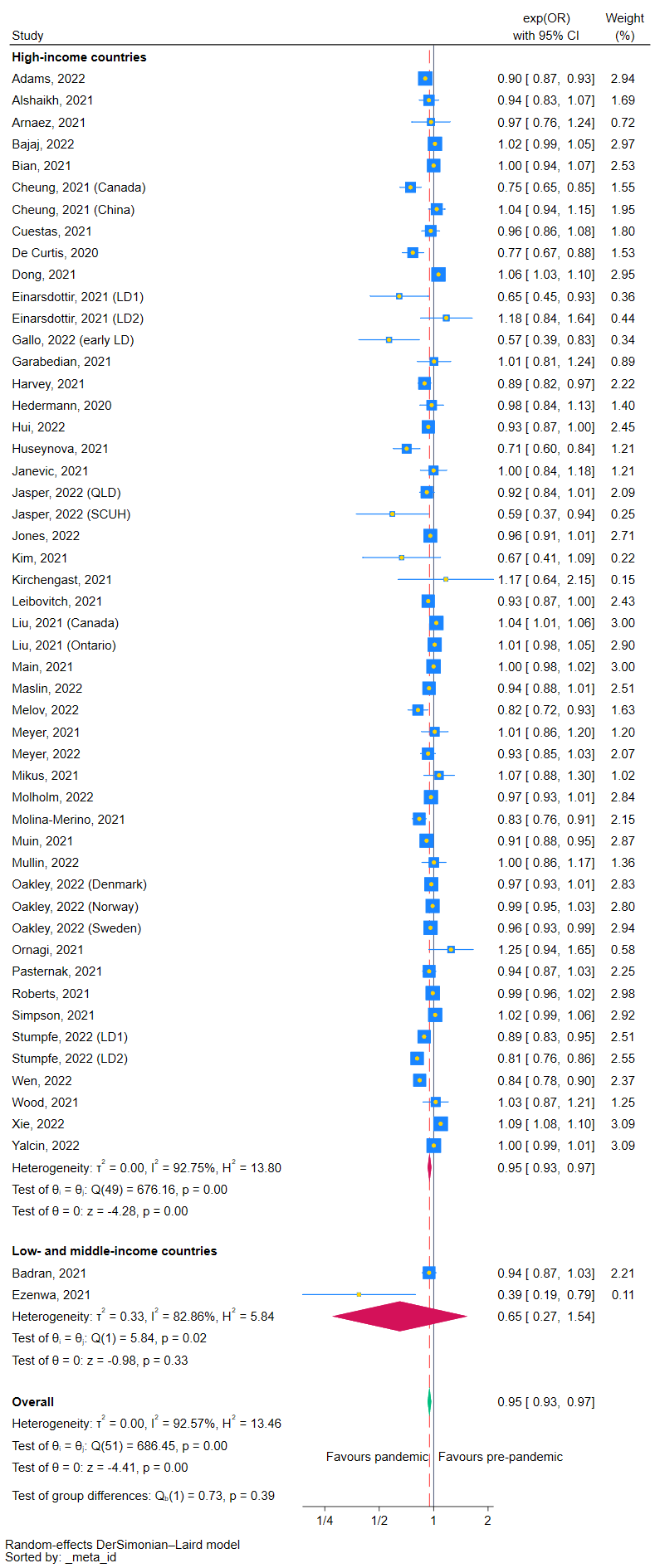


**Supplementary Figure 4 Rates of preterm birth in the pre-pandemic and pandemic periods according to stringency of mitigation measures**

**
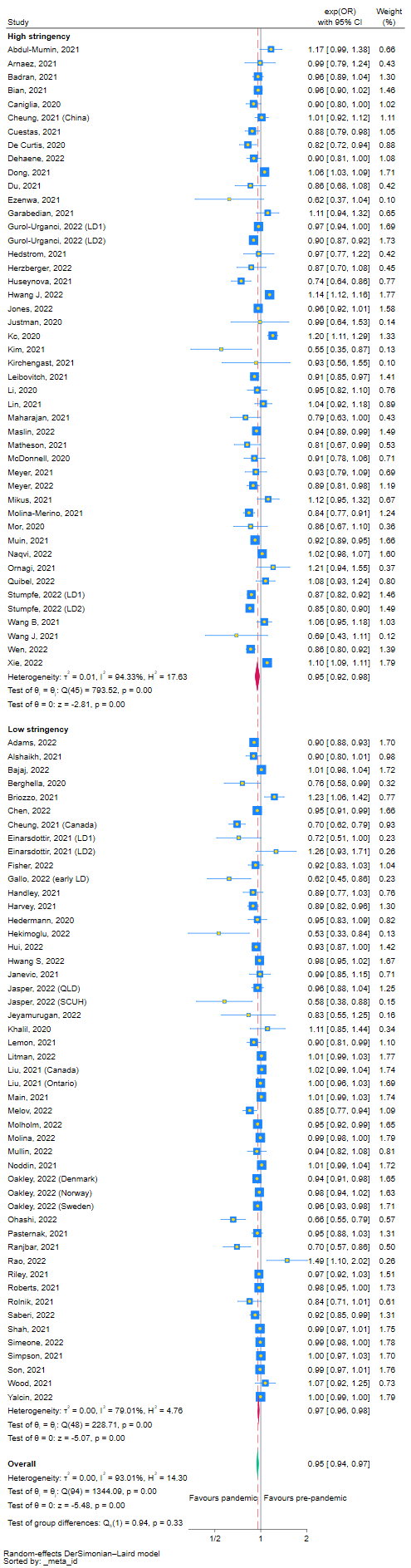
**

**Supplementary Figure 5a Rates of spontaneous preterm birth in the pre-pandemic and pandemic periods according to World Bank Classification**


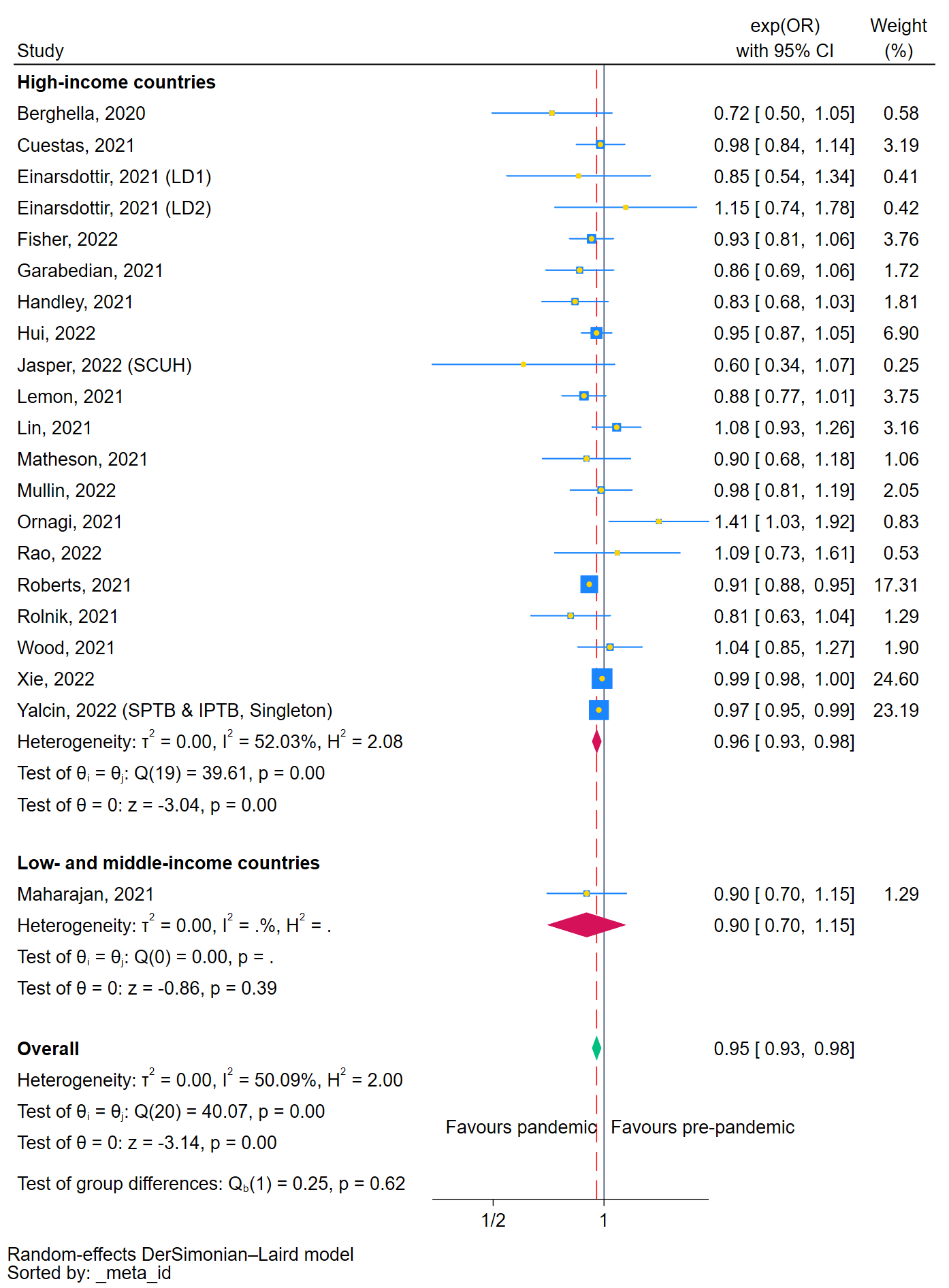


**Supplementary Figure 5b Rates of spontaneous preterm birth in the pre-pandemic and pandemic periods according to stringency of mitigation measures**

**
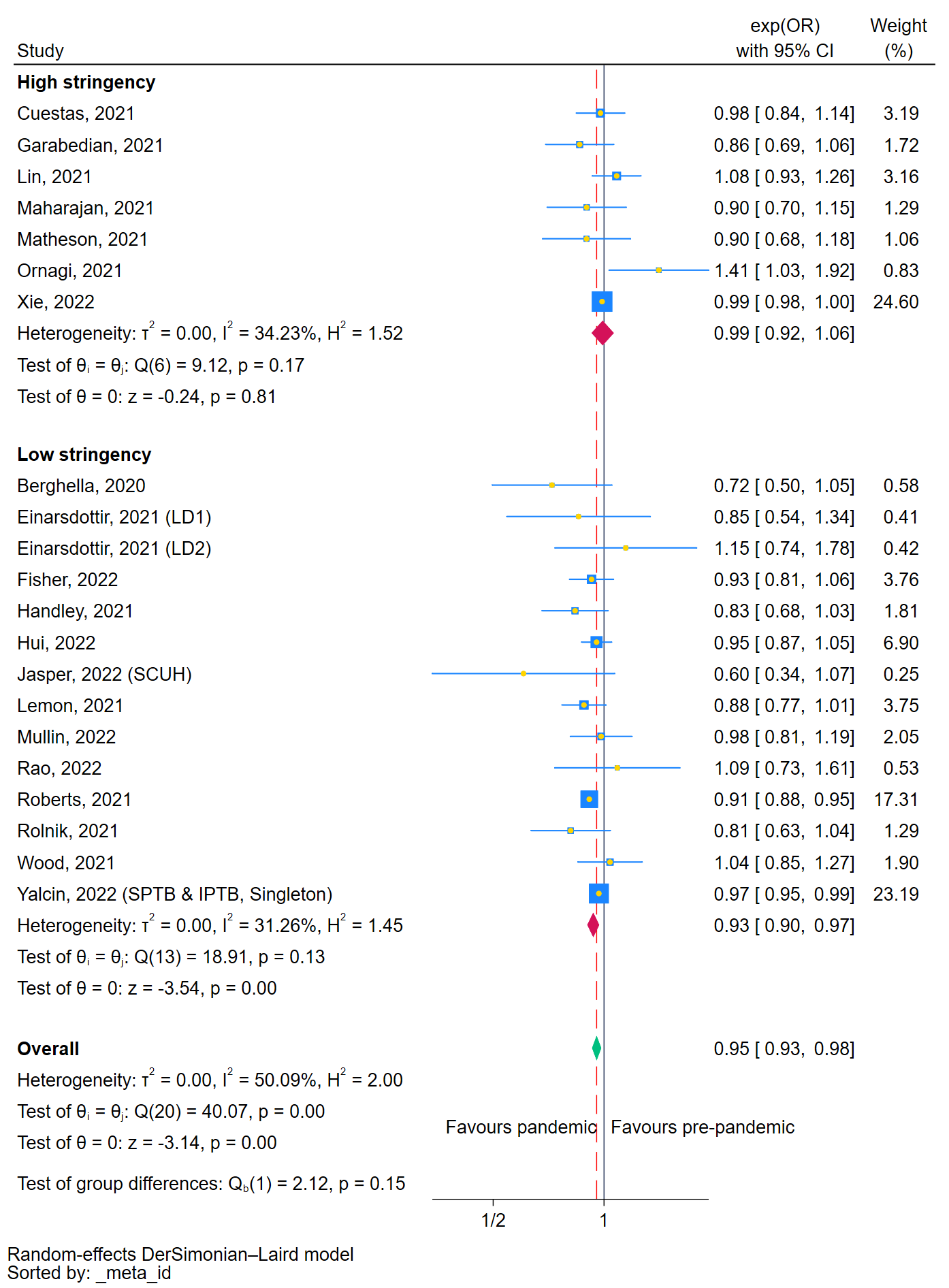
**

**Supplementary Figure 5c Rates of medically indicated preterm birth in the pre-pandemic and pandemic periods according to World Bank Classification**


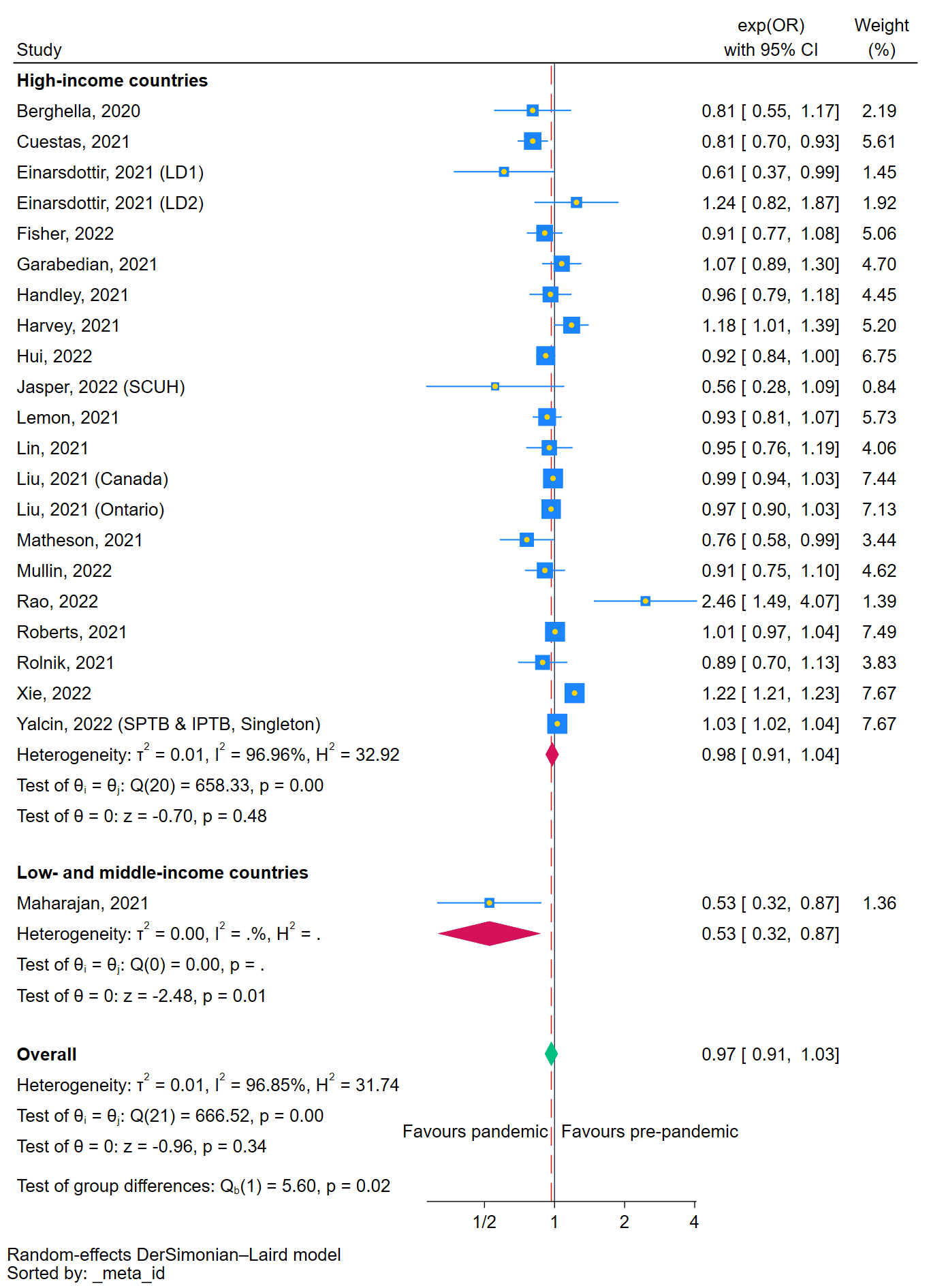


**Supplementary Figure 6a Rates of low birth weight (<2500g) in the pre-pandemic and pandemic periods according to World Bank Classification**


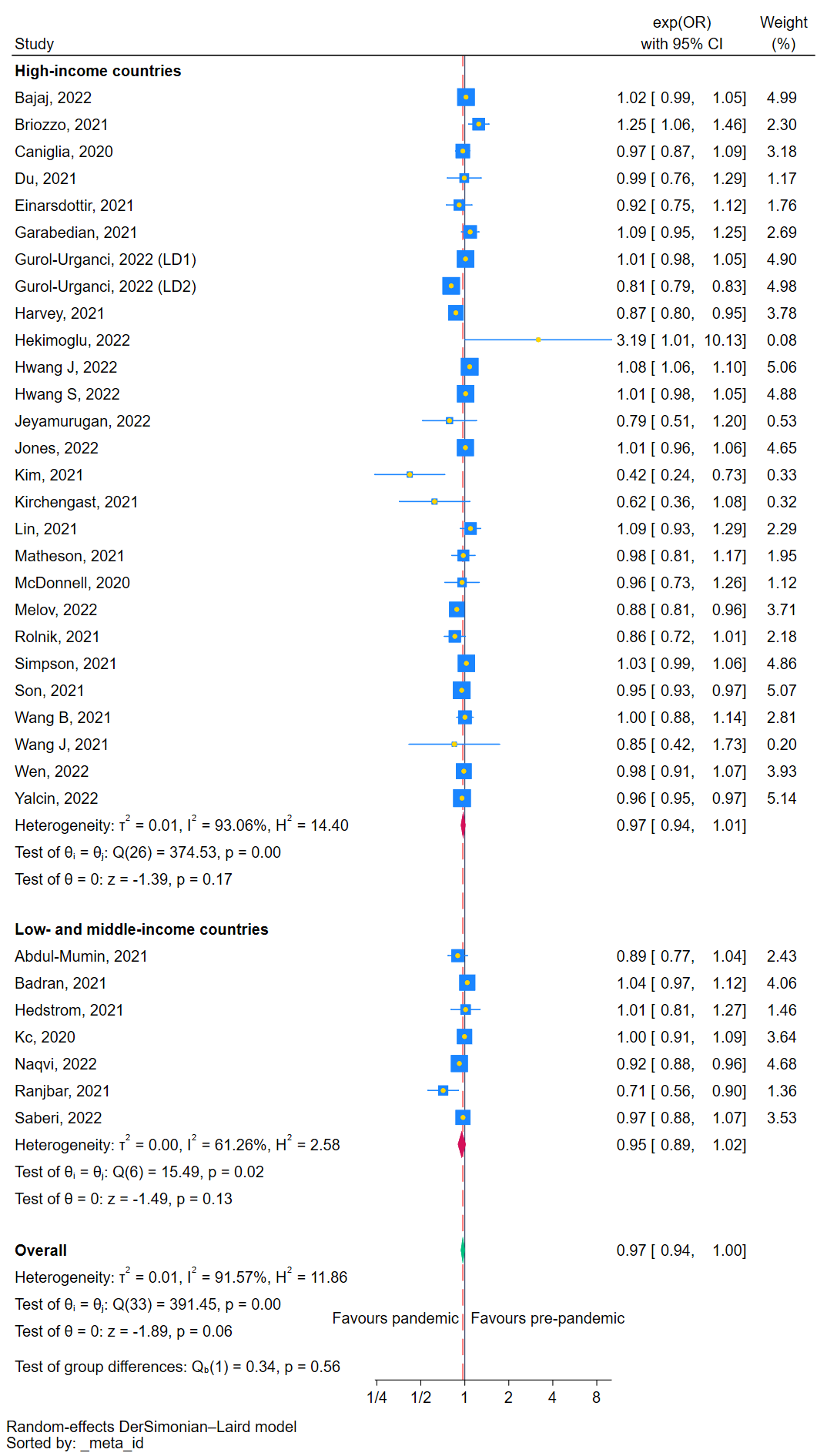


**Supplementary Figure 6b Rates of very low birth weight (<1500g) in the pre-pandemic and pandemic periods according to World Bank Classification**


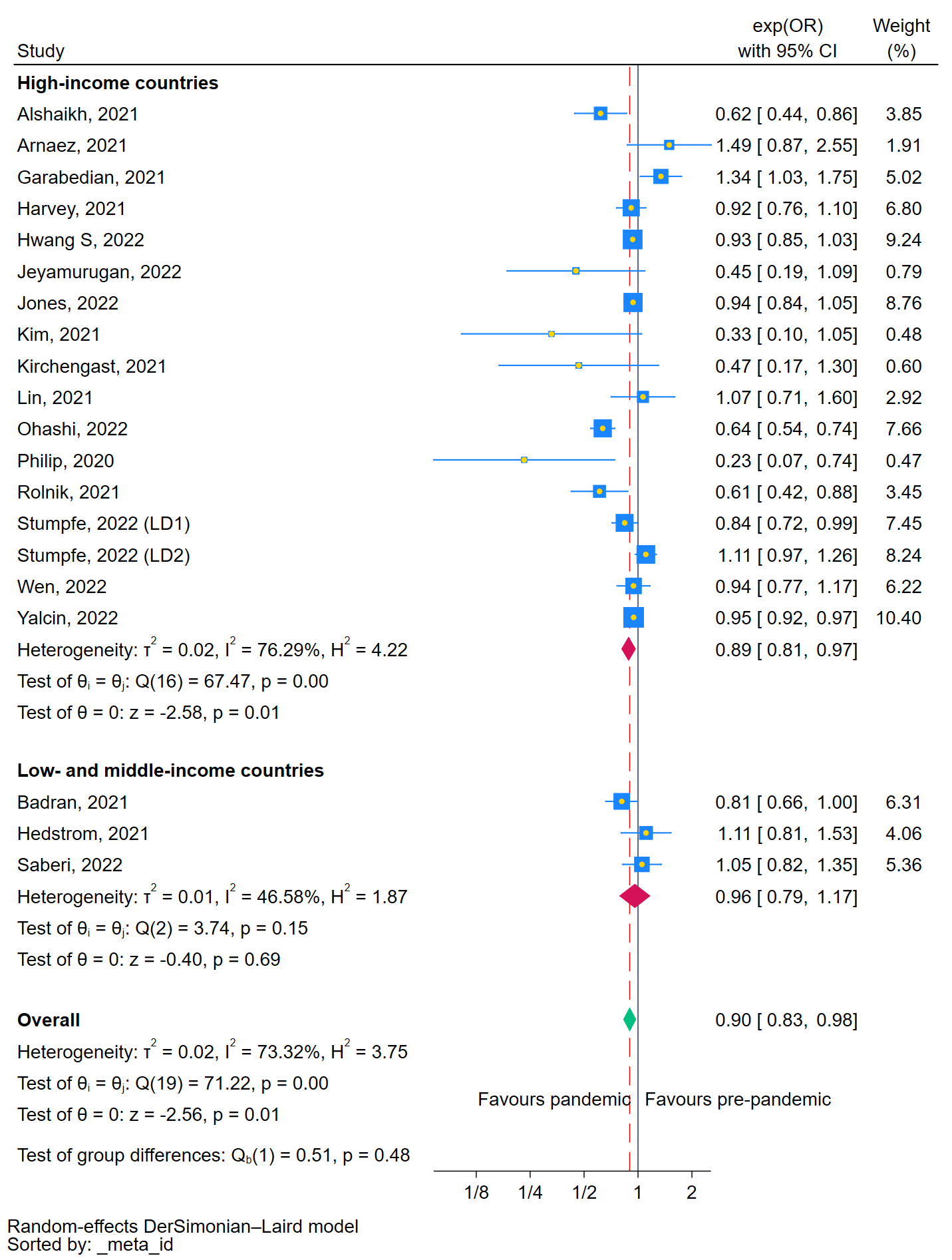


**Supplementary Figure 7 Rates of stillbirths in the pre-pandemic and pandemic periods according to stringency of mitigation measures**


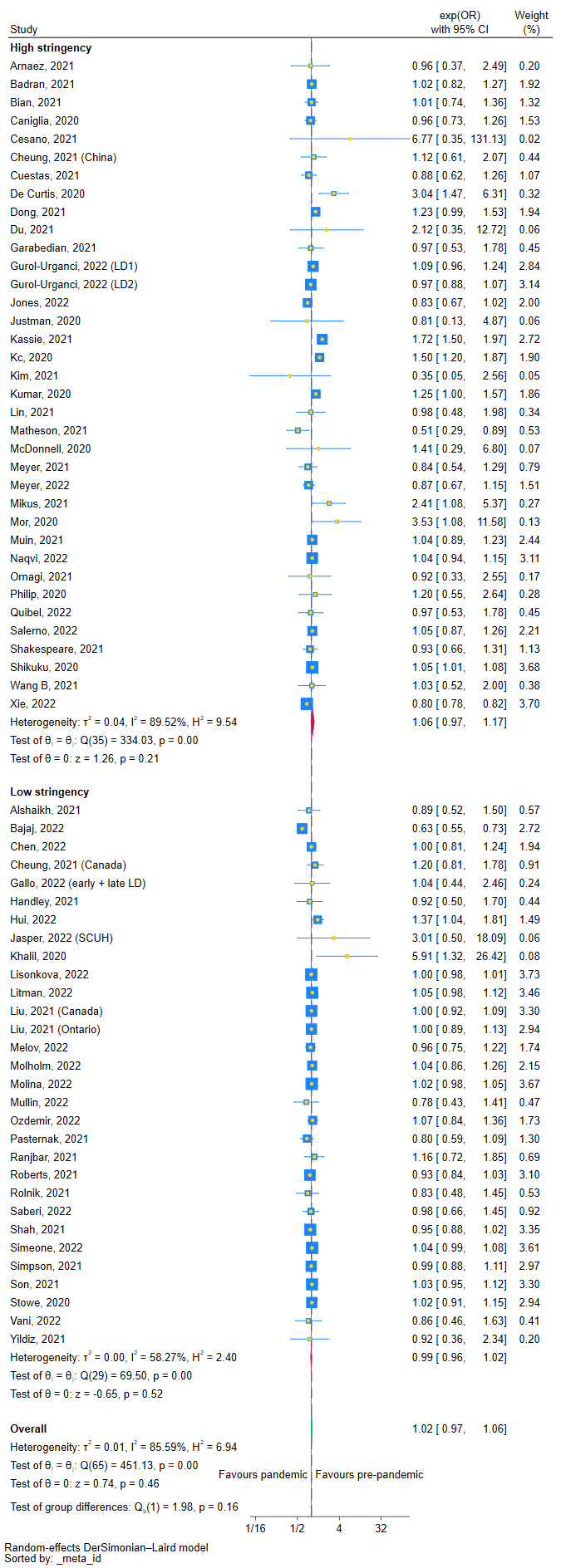


**Supplementary Figure 8 Rates of neonatal death in the pre-pandemic and pandemic periods according to stringency of mitigation measures**


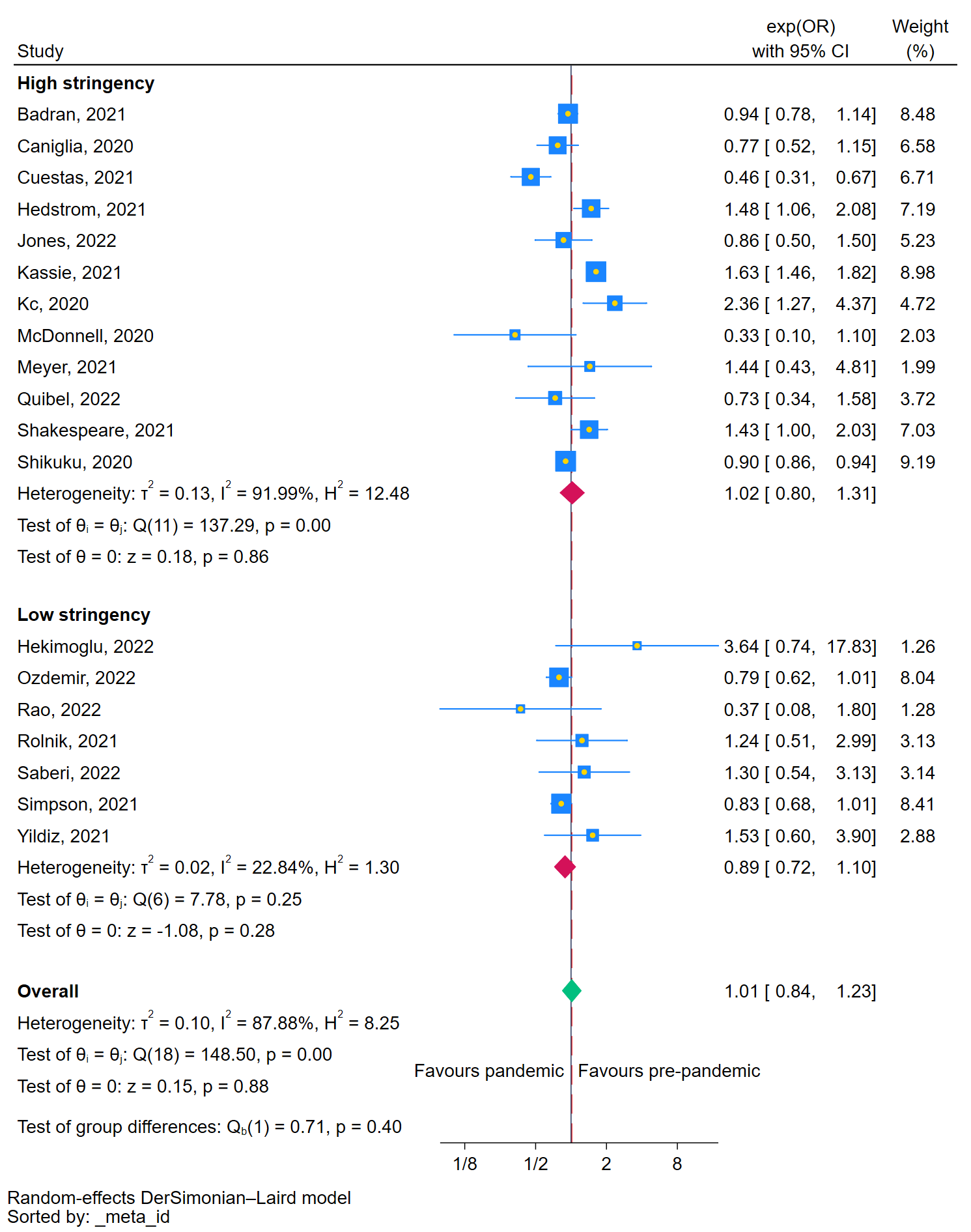


**Supplementary Figure 9a Rates of caesarean section in the pre-pandemic and pandemic periods according to World Bank Classification**


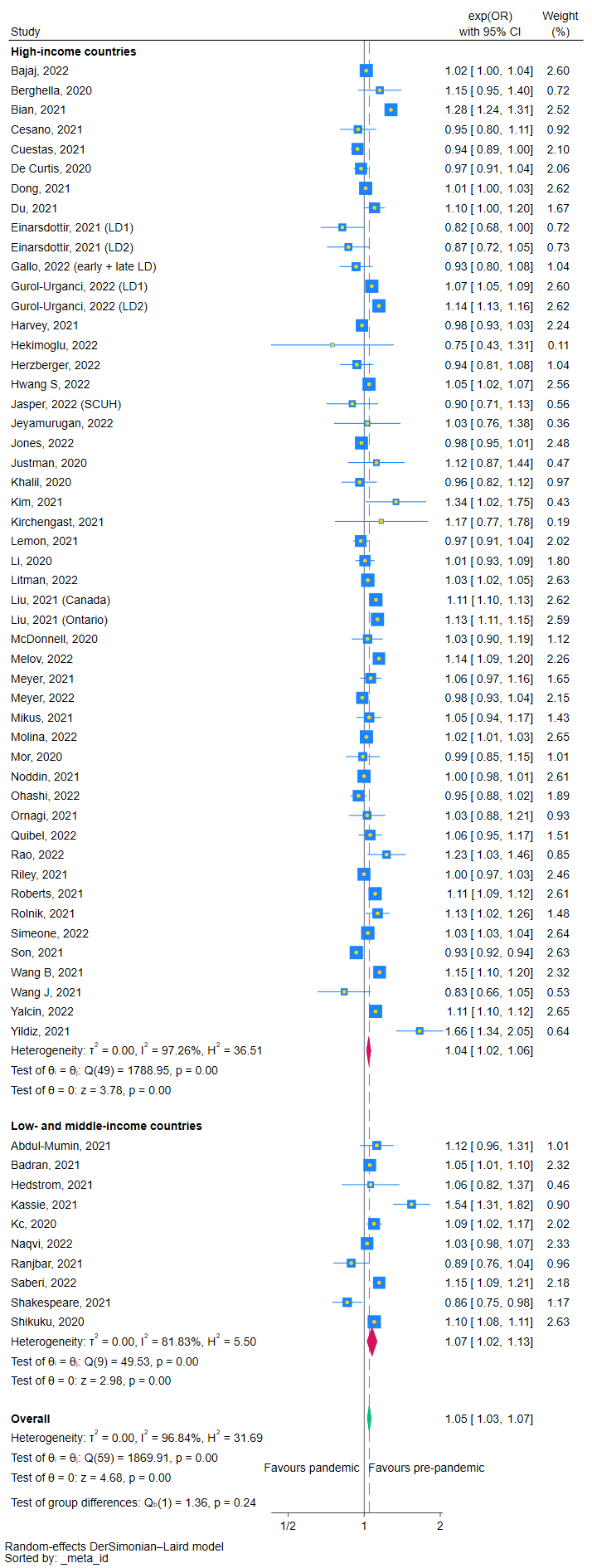


**Supplementary Figure 9b Rates of caesarean section in the pre-pandemic and pandemic periods according to stringency of mitigation measures**


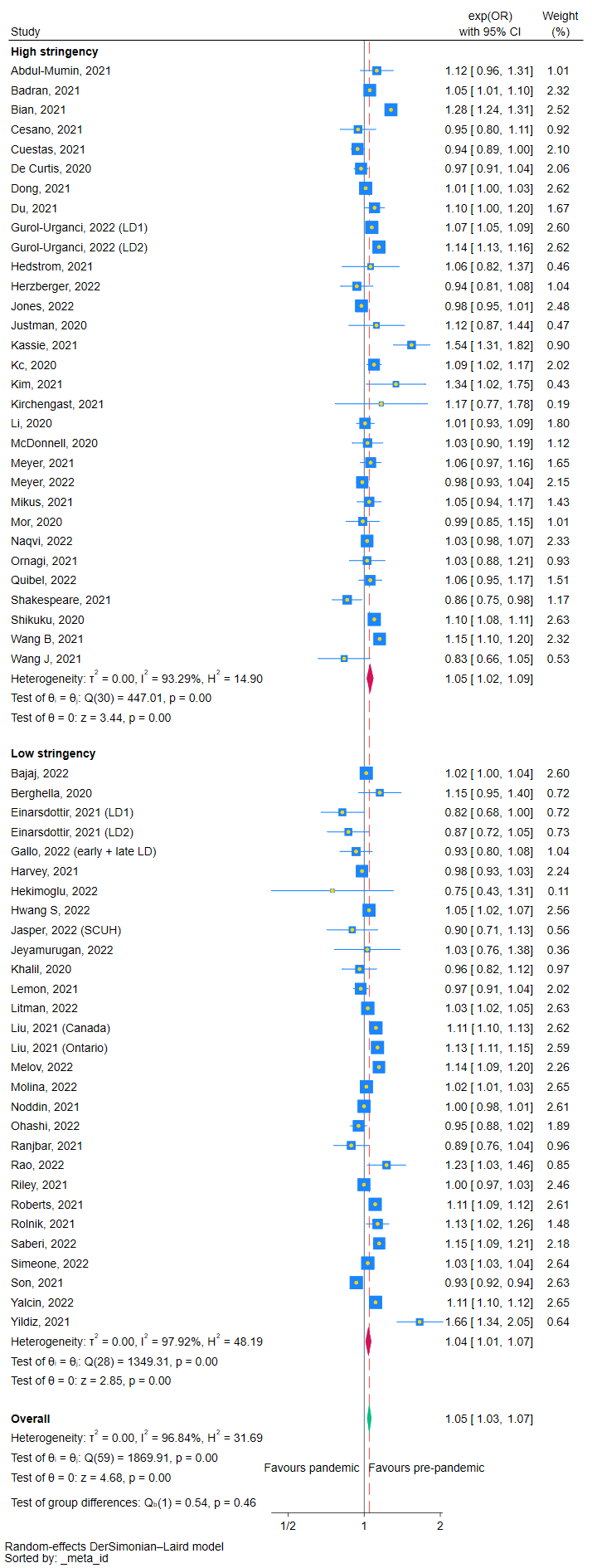


**Supplementary references**

1. Abdul-Mumin A, Cotache-Condor C, Bimpong KA, Grimm A, Kpiniong MJ; Yakubu RC, Kwarteng PG, et al. Decrease in admissions and change in the diagnostic landscape in a newborn care unit in Northern Ghana during the COVID-19 pandemic. *Front Pediatr*. 2021;9(101615492):642508

2. Adams M, Schulzke SM, Rogdo B, Meyer P, McDougall J, Stocker M, Schneider J, et al, Swiss Neonatal Network. Impact of SARS-CoV-2 on incidence, treatment and outcome of very preterm born infants in Switzerland: a retrospective, population-based cohort study. *Swiss Med Wkly*. 2022;152:30174.

3. Alshaikh B, Cheung PY, Soliman N, Brundler MA, Yusuf K. Impact of Lockdown Measures during COVID-19 Pandemic on Pregnancy and Preterm Birth. *Am J Perinatol*. 2022;39(3):329-336.

4. Arnaez J, Ochoa-Sangrador C, Caserío S, Gutiérrez EP, Jiménez MDP, Castañón L, Benito M, et al. Lack of changes in preterm delivery and stillbirths during COVID-19 lockdown in a European region. *Eur J Pediatr*. 2021;180(6):1997-2002.

5. Badran EF, Darwish RM, Khader Y, AlMasri R, Al Jaberi M, AlMasri M, AlSa'di F, et al. Adverse pregnancy outcomes during the COVID-19 lockdown. A descriptive study. *BMC Pregnancy Childbirth*. 2021;21(1):761.

6. Bajaj M, Romero R, Myers L, Duncan J, Yeo L, Jani S, Natarajan G. Population-Level Study on Fetal Deaths and Preterm Births during SARS-CoV-2 Pandemic in the State of Michigan. *Am J Perinatol*. 2024;41(S 01):e236-e248.

7. Berghella V, Boelig R, Roman A, Burd J, Anderson K. Decreased incidence of preterm birth during coronavirus disease 2019 pandemic. *Am J Obstet Gynecol MFM*. 2020;2(4):100258.

8. Bian Z, Qu X, Ying H, Liu X. Are COVID-19 mitigation measures reducing preterm birth rate in China? *BMJ Glob Health*. 2021;6(8):e006359.

9. Briozzo L, Tomasso G, Viroga S, Nozar F, Bianchi A. Impact of mitigation measures against the COVID 19 pandemic on the perinatal results of the reference maternity hospital in Uruguay. *J Matern Fetal Neonatal Med*. 2022;35(25):5060-5062.
10. Caniglia EC, Magosi LE, Zash R, Diseko M, Mayondi G, Mabuta J, Powis K, et al. Modest reduction in adverse birth outcomes following the COVID-19 lockdown. *Am J Obstet Gynecol*. 2021;224(6):615.e1-615.e12.

11. Cesano N, D'Ambrosi F, Cetera GE, Carbone IF, Di Maso M, Ossola MW, Iurlaro E, et al. Maternity ward management and COVID-19 pandemic: Experience of a single center in Northern Italy during lockdown. *Eur J Midwifery*. 2021;5:29.

12. Chen J, Ferre C, Ouyang L, Mohamoud Y, Barfield W, Cox S. Changes and geographic variation in rates of preterm birth and stillbirth during the prepandemic period and COVID-19 pandemic, according to health insurance claims in the United States, April-June 2019 and April-June 2020. *Am J Obstet Gynecol MFM*. 2022;4(1):100508.

13. Cheung PY, Alshaikh B, Yang C. COVID-19 Pandemic: Different Associative Relationships of City Lockdown With Preterm Births in Three Cities - An Ecological Study. *Front Pediatr*. 2021;9:644771.

14. Cuestas E, Gómez-Flores ME, Charras MD, Peyrano AJ, Montenegro C, Sosa-Boye I, Burgos V, et al. Association between COVID-19 mandatory lockdown and decreased incidence of preterm births and neonatal mortality. *J Perinatol*. 2021;41(10):2566-2569.

15. De Curtis M, Villani L, Polo A. Increase of stillbirth and decrease of late preterm infants during the COVID-19 pandemic lockdown. *Arch Dis Child Fetal Neonatal Ed*. 2021;106(4):456.

16. Dehaene I, Van Holsbeke C, Roelens K, van Oostrum NN, Nulens K, Smets K, Van Hoestenberghe MR, et al. Preterm birth during the COVID-19 pandemic: more, less, or just the same? *Acta Clin Belg*. 2023;78(2):140-159.

17. Dong M, Qian R, Wang J, Fan J, Ye Y, Zhou H, Win B, et al. Associations of COVID-19 lockdown with gestational length and preterm birth in China. *BMC Pregnancy Childbirth*. 2021;21(1):795.

18. Du M, Yang J, Han N, Liu M, Liu J. Association between the COVID-19 pandemic and the risk for adverse pregnancy outcomes: a cohort study. *BMJ Open*. 2021;11(2):e047900.

19. Einarsdóttir K, Swift EM, Zoega H. Changes in obstetric interventions and preterm birth during COVID-19: A nationwide study from Iceland. *Acta Obstet Gynecol Scand*. 2021;100(10):1924-1930

20. Ezenwa BN, Fajolu IB, Nabwera H, Wang D, Ezeaka CV, Allen S. Impact of COVID-19 lockdown measures on institutional delivery, neonatal admissions and prematurity: a reflection from Lagos, Nigeria. *BMJ Paediatr Open*. 2021;5(1):e001029.

21. Fisher SA, Sakowicz A, Barnard C, Kidder S, Miller ES. Neighborhood deprivation and preterm delivery during the coronavirus 2019 pandemic. *Am J Obstet Gynecol MFM*. 2022;4(1):100493.

22. Gallo LA, Gallo TF, Borg DJ, Moritz KM, Clifton VL, Kumar S. A decline in planned, but not spontaneous, preterm birth rates in a large Australian tertiary maternity centre during COVID-19 mitigation measures. *Aust N Z J Obstet Gynaecol.* 2022;62(1):62-70.

23. Garabedian C, Dupuis N, Vayssière C, Bussières L, Ville Y, Renaudin B, Dugave L, et al. Impact of COVID-19 Lockdown on Preterm Births, Low Birthweights and Stillbirths: A Retrospective Cohort Study. *J Clin Med*. 2021;10(23):5649.

24. Gurol-Urganci I, Waite L, Webster K, Jardine J, Carroll F, Dunn G, Frémeaux A, et al. Obstetric interventions and pregnancy outcomes during the COVID-19 pandemic in England: A nationwide cohort study. *PLoS Med*. 2022;19(1):e1003884.

25. Handley SC, Mullin AM, Elovitz MA, Gerson KD, Montoya-Williams D, Lorch SA, Burris HH. Changes in Preterm Birth Phenotypes and Stillbirth at 2 Philadelphia Hospitals During the SARS-CoV-2 Pandemic, March-June 2020. *JAMA*. 2021;325(1):87-89.

26. Harvey EM, McNeer E, McDonald MF, Shapiro-Mendoza CK, Dupont WD, Barfield W, Patrick SW. Association of Preterm Birth Rate With COVID-19 Statewide Stay-at-Home Orders in Tennessee. *JAMA Pediatr.* 2021;175(6):635-637.

27. Hedermann G, Hedley PL, Bækvad-Hansen M, Hjalgrim H, Rostgaard K, Poorisrisak P, Breindahl M, et al. Danish premature birth rates during the COVID-19 lockdown. *Arch Dis Child Fetal Neonatal Ed*. 2021;106(1):93-95.

28. Hedley PL, Hedermann G, Hagen CM, Bækvad-Hansen M, Hjalgrim H, Rostgaard K, Laksafoss AD, et al. Preterm birth, stillbirth and early neonatal mortality during the Danish COVID-19 lockdown. *Eur J Pediatr*. 2022;181(3):1175-1184

29. Hedstrom A, Mubiri P, Nyonyintono J, Nakakande J, Magnusson B, Vaughan M, Waiswa P, et al. Impact of the early COVID-19 pandemic on outcomes in a rural Ugandan neonatal unit: A retrospective cohort study. *PLoS One*. 2021;16(12):e0260006.

30. Hekimoğlu B, Aktürk Acar F. Effects of COVID-19 pandemic period on neonatal mortality and morbidity. *Pediatr Neonatol*. 2022;63(1):78-83.

31. Herzberger E, Efros O, Herzberger S, Biron-Shental T, Shechter-Maor G. Differences in obstetric healthcare utilization and delivery complications before and after the COVID-19 pandemic - a retrospective study*. J Matern Fetal Neonatal Med*. 2022;35(25):7194-7199.

32. Hui L, Marzan MB, Potenza S, Rolnik DL, Pritchard N, Said JM, Palmer KR, et al. Increase in preterm stillbirths in association with reduction in iatrogenic preterm births during COVID-19 lockdown in Australia: a multicenter cohort study. *Am J Obstet Gynecol*. 2022;227(3):491.e1-491.e17.

33. Huseynova R, Bin Mahmoud L, Abdelrahim A, Al Hemaid M, Almuhaini MS, Jaganathan PP, Career H, et al. Prevalence of Preterm Birth Rate During COVID-19 Lockdown in a Tertiary Care Hospital, Riyadh. *Cureus*. 2021;13(3):e13634.

34. Hwang J, Moon S, Cho KD, Oh MJ, Hong SJ, Cho GJ. Changes in preterm birth and birthweight during the SARS-CoV-2 pandemic: a nationwide study in South Korea. *Sci Rep*. 2022;12(1):16288.

35. Hwang SS, Weikel BW, Hannan KE, Bourque SL. Impact of Coronavirus Disease-19 "Stay-At-Home" Orders on Preterm Birth in Colorado. *J Pediatr*. 2022;242:238-241.e1.

36. Janevic T, Glazer KB, Vieira L, Weber E, Stone J, Stern T, Bianco A, et al. Racial/Ethnic Disparities in Very Preterm Birth and Preterm Birth Before and During the COVID-19 Pandemic. *JAMA Netw Open*. 2021;4(3):e211816.

37. Jasper B, Stillerova T, Anstey C, Weaver E. Reduction in preterm birth rates during and after the COVID-19 lockdown in Queensland Australia. *Aust N Z J Obstet Gynaecol*. 2022;62(6):851-858.

38. Jeyamurugan K, Jung MK, Ericksen K. Preterm birth rates in a deprived American community only differed before and during COVID-19 when maternal risks were factored in. *Acta Paediatr*. 2023;112(1):151-153.

39. Jones H, Seaborne M, Cowley L, Odd D, Paranjothy S, Akbari A, Brophy S. Population birth outcomes in 2020 and experiences of expectant mothers during the COVID-19 pandemic: A 'born in Wales' mixed methods study using routine data. *PLoS One*. 2022;17(5):e0267176.

40. Justman N, Shahak G, Gutzeit O, Ben Zvi D, Ginsberg Y, Solt I, Vitner D, et al. Lockdown with a Price: The impact of the COVID-19 Pandemic on Prenatal Care and Perinatal Outcomes in a Tertiary Care Center. *Isr Med Assoc J*. 2020;22(9):533-537.

41. Kassie A, Wale A, Yismaw W. Impact of Coronavirus Diseases-2019 (COVID-19) on Utilization and Outcome of Reproductive, Maternal, and Newborn Health Services at Governmental Health Facilities in South West Ethiopia, 2020: Comparative Cross-Sectional Study. *Int J Womens Health*. 2021;13:479-488.

42. KC A, Gurung R, Kinney MV, Sunny AK, Moinuddin M, Basnet O, Paudel P, et al. Effect of the COVID-19 pandemic response on intrapartum care, stillbirth, and neonatal mortality outcomes in Nepal: a prospective observational study. *Lancet Glob Health.* 2020;8(10):e1273-1281.

43. Khalil A, von Dadelszen P, Draycott T, Ugwumadu A, O'Brien P, Magee L. Change in the Incidence of Stillbirth and Preterm Delivery During the COVID-19 Pandemic. *JAMA*. 2020;324(7):705–6.

44. Kim SY, Kim SY, Kil K, Lee Y. Impact of COVID-19 Mitigation Policy in South Korea on the Reduction of Preterm or Low Birth Weight Birth Rate: A Single Center Experience. *Children (Basel).* 2021;8(5):332.

45. Kirchengast S, Hartmann B. Pregnancy Outcome during the First COVID 19 Lockdown in Vienna, Austria. *Int J Environ Res Public Health*. 2021;18(7):3782.

46. Klumper J, Kazemier BM, Been JV, Bloemenkamp KWM, de Boer MA, Erwich JJHM, Heidema W, et al. Association between COVID-19 lockdown measures and the incidence of iatrogenic versus spontaneous very preterm births in the Netherlands: a retrospective study. *BMC Pregnancy Childbirth*. 2021;21(1):767.

47. Kumar M, Puri M, Yadav R, Biswas R, Singh M, Chaudhary V, Jaiswal N, et al. Stillbirths and the COVID-19 pandemic: Looking beyond SARS-CoV-2 infection. *Int J Gynaecol Obstet*. 2021;153(1):76-82.

48. Leibovitch L, Reichman B, Mimouni F, Zaslavsky-Paltiel I, Lerner-Geva L, Wasserteil N, Sagiv N, et al. Preterm Singleton Birth Rate during the COVID-19 Lockdown: A Population-Based Study. *Am J Perinatol.* 2022;39(9):1020-1026.

49. Lemon L, Edwards RP, Simhan HN. What is driving the decreased incidence of preterm birth during the coronavirus disease 2019 pandemic? *Am J Obstet Gynecol MFM*. 2021;3(3):100330.

50. Lin TT, Zhang C, Chen L, Jin L, Lin XH, Pan JX, Dennis CL, et al. COVID-19 Lockdown Increased the Risk of Preterm Birth. *Front Med (Lausanne).* 2021;8:705943.

51. Lisonkova S, Bone JN, Muraca GM, Razaz N, Boutin A, Brandt JS, Bedaiwy MA, et al. Early coronavirus disease 2019 restrictive measures and changes in maternal characteristics, use of assisted reproductive technology, and stillbirth. *Paediatr Perinat Epidemiol*. 2023;37(2):117-127.

52. Litman EA, Yin Y, Nelson SJ, Capbarat E, Kerchner D, Ahmadzia HK. Adverse perinatal outcomes in a large United States birth cohort during the COVID-19 pandemic. *Am J Obstet Gynecol MFM.* 2022;4(3):100577.

53. Liu S, Dzakpasu S, Nelson C, Wei SQ, Little J, Scott H, Joseph KS. Pregnancy Outcomes During the COVID-19 Pandemic in Canada, March to August 2020. *J Obstet Gynaecol Can*. 2021;43(12):1406-1415.

54. Mahajan NN, Pednekar R, Gaikwad C, More P, Pophalkar M, Kesarwani S, Jnanananda B, Mahale SD, Gajbhiye RK. Increased spontaneous preterm births during the second wave of the coronavirus disease 2019 pandemic in India. *Int J Gynaecol Obstet*. 2022;157(1):115-120.

55. Main EK, Chang SC, Carpenter AM, Wise PH, Stevenson DK, Shaw GM, Gould JB. Singleton preterm birth rates for racial and ethnic groups during the coronavirus disease 2019 pandemic in California. *Am J Obstet Gynecol*. 2021;224(2):239-241.

56. Maslin K, McKeon-Carter R, Hosking J, Stockley L, Southby C, Shawe J, Latour JM. Preterm births in South-West England before and during the COVID-19 pandemic: an audit of retrospective data. *Eur J Pediatr.* 2022;181(2):859-863.

57. Matheson A, McGannon CJ, Malhotra A, Palmer KR, Stewart AE, Wallace EM, Mol BW, et al. Prematurity Rates During the Coronavirus Disease 2019 (COVID-19) Pandemic Lockdown in Melbourne, Australia. *Obstet Gynecol.* 2021;137(3):405-407.

58. McDonnell S, McNamee E, Lindow SW, O'Connell MP. The impact of the Covid-19 pandemic on maternity services: A review of maternal and neonatal outcomes before, during and after the pandemic. *Eur J Obstet Gynecol Reprod Biol*. 2020;255:172-176.

59. Melov SJ, Elhindi J, McGee TM, Lee VW, Cheung NW, Chua SC, McNab J, et al. Investigating service delivery and perinatal outcomes during the low prevalence first year of COVID-19 in a multiethnic Australian population: a cohort study. *BMJ Open*. 2022;12(7):e062409.

60. Meyer R, Bart Y, Tsur A, Yinon Y, Friedrich L, Maixner N, Levin G. A marked decrease in preterm deliveries during the coronavirus disease 2019 pandemic. *Am J Obstet Gynecol*. 2021;224(2):234-237.

61. Meyer R, Friedrich L, Levin G. Low Covid-19 infection rate period is associated with a rebound increase in preterm birth rate. *J Perinatol*. 2023;43(5):670-672.

62. Mikuš M, Sokol Karadjole V, Kalafatić D, Orešković S, Šarčević A. Increase of stillbirths and unplanned out-of-hospital births during coronavirus disease 2019 lockdown and the Zagreb earthquake. *Acta Obstet Gynecol Scand*. 2021;100(11):2119-2120.

63. Mølholm Hansen B, Cueto H, Padkaer Petersen J, Zachariassen G, Sønderby Christensen P, Breindahl M, et al. Preterm birth rates were slightly lower in Denmark during the first year of the COVID-19 pandemic compared with the previous 4 years. *Acta Paediatr*. 2022;111(9):1695-1700.

64. Molina RL, Tsai TC, Dai D, Soto M, Rosenthal N, Orav EJ, Figueroa JF. Comparison of Pregnancy and Birth Outcomes Before vs During the COVID-19 Pandemic. *JAMA Netw Open*. 2022;5(8):e2226531.

65. Molina-Merino A, Martínez-Rodríguez L, Cubells-García E, Hortelano-Platero V, Estañ-Capell J. Effect of lockdown on the preterm births in a Valencian Comunity. *An Pediatr (Engl Ed).* 2021;95(5):379-381.

66. Mor M, Kugler N, Jauniaux E, Betser M, Wiener Y, Cuckle H, Maymon R. Impact of the COVID-19 Pandemic on Excess Perinatal Mortality and Morbidity in Israel. *Am J Perinatol.* 2021;38(4):398-403.

67. Muin DA, Neururer S, Falcone V, Windsperger K, Helmer H, Leitner H, Kiss H, et al. Antepartum stillbirth rates during the COVID-19 pandemic in Austria: A population-based study. *Int J Gynaecol Obstet.* 2022;156(3):459-465.

68. Mullin AM, Handley SC, Lundsberg L, Elovitz MA, Lorch SA, McComb EJ, Montoya-Williams D, et al. Changes in preterm birth during the COVID-19 pandemic by duration of exposure and race and ethnicity. *J Perinatol.* 2022;42(10):1346-1352.

69. Naqvi S, Naqvi F, Saleem S, Thorsten VR, Figueroa L, Mazariegos M, Garces A, et al. Health care in pregnancy during the COVID-19 pandemic and pregnancy outcomes in six low- and-middle-income countries: Evidence from a prospective, observational registry of the Global Network for Women's and Children's Health. *BJOG*. 2022;129(8):1298-1307.

70. Noddin K, Bradley D, Wolfberg A. Delivery Outcomes During the COVID-19 Pandemic as Reported in a Pregnancy Mobile App: Retrospective Cohort Study. *JMIR Pediatr Parent*. 2021;4(4):e27769.

71. Oakley LL, Örtqvist AK, Kinge J, Hansen AV, Petersen TG, Söderling J, Telle KE, et al. Preterm birth after the introduction of COVID-19 mitigation measures in Norway, Sweden, and Denmark: a registry-based difference-in-differences study. *Am J Obstet Gynecol*. 2022;226(4):550.e1-550.e22.

72. Ohashi M, Tsuji S, Tanaka-Mizuno S, Kasahara K, Kasahara M, Miura K, Murakami T. Amelioration of prevalence of threatened preterm labor during the COVID-19 pandemic: nationwide database analysis in Japan. *Sci Rep*. 2022;12(1):15345.

73. Ornaghi S, Fumagalli S, Guinea Montalvo CK, Beretta G, Invernizzi F, Nespoli A, Vergani P. Indirect impact of SARS-CoV-2 pandemic on pregnancy and childbirth outcomes: A nine-month long experience from a university center in Lombardy. *Int J Gynaecol Obstet.* 2022;156(3):466-474.

74. Ozdemir S, Oruç MA. Evaluation of stillbirths and infant mortality before and during the COVID-19 pandemic: a retrospective study. *Postgrad Med*. 2022;134(5):524-532.

75. Pasternak B, Neovius M, Söderling J, Ahlberg M, Norman M, Ludvigsson JF, Stephansson O. Preterm Birth and Stillbirth During the COVID-19 Pandemic in Sweden: A Nationwide Cohort Study. *Ann Intern Med.* 2021;174(6):873-875.

76. Philip RK, Purtill H, Reidy E, Daly M, Imcha M, McGrath D, O'Connell NH, et al. Unprecedented reduction in births of very low birthweight (VLBW) and extremely low birthweight (ELBW) infants during the COVID-19 lockdown in Ireland: a 'natural experiment' allowing analysis of data from the prior two decades. *BMJ Glob Health*. 2020;5(9):e003075.

77. Quibel T, Winer N, Bussières L, Vayssière C, Deruelle P, Defrance M, Rozenberg P, et al. Impact of COVID-19-Related Lockdown on Delivery and Perinatal Outcomes: A Retrospective Cohort Study. *J Clin Med*. 2022;11(3):756.

78. Ranjbar F, Allahqoli L, Ahmadi S, Mousavi R, Gharacheh M, Eshraghi N, Alkatout I. Changes in pregnancy outcomes during the COVID-19 lockdown in Iran. *BMC Pregnancy Childbirth*. 2021;21(1):577.

79. Rao MG, Toner LE, Stone J, Iwelumo CA, Goldberger C, Roser BJ, Shah R, et al. Pregnancy during a Pandemic: A Cohort Study Comparing Adverse Outcomes during and before the COVID-19 Pandemic. *Am J Perinatol*. 2023;40(4):445-452.

80. Riley T, Nethery E, Chung EK, Souter V. Impact of the COVID-19 pandemic on perinatal care and outcomes in the United States: An interrupted time series analysis. *Birth*. 2022;49(2):298-309.

81. Roberts NF, Sprague AE, Taljaard M, Fell DB, Ray JG, Tunde-Byass M, Biringer A, Barrett JFR, et al. Maternal-Newborn Health System Changes and Outcomes in Ontario, Canada, During Wave 1 of the COVID-19 Pandemic-A Retrospective Study. *J Obstet Gynaecol Can*. 2022;44(6):664-674.

82. Rolnik DL, Matheson A, Liu Y, Chu S, Mcgannon C, Mulcahy B, Malhotra A, et al. Impact of COVID-19 pandemic restrictions on pregnancy duration and outcome in Melbourne, Australia. *Ultrasound Obstet Gynecol.* 2021;58(5):677-687.

83. Saberi H, Ghorashi Z, Loripoor M. Comparison of pregnancy outcome during pandemic of COVID-19 and non-pandemic situations, Yazd, Iran, in 2019-2020. *J Obstet Gynaecol.* 2022;42(6):1937-1943.

84. Salerno C, Donno V, Melis B, Perrone E, Menichini D, Facchinetti F, Monari F. Stillbirth occurrence during COVID-19 pandemic: a population-based prospective study*. J Perinat Med*. 2022;50(6):653-659.

85. Shah PS, Ye XY, Yang J, Campitelli MA. Preterm birth and stillbirth rates during the COVID-19 pandemic: a population-based cohort study. *CMAJ*. 2021;193(30):E1164-E1172.

86. Shakespeare C, Dube H, Moyo S, Ngwenya S. Resilience and vulnerability of maternity services in Zimbabwe: a comparative analysis of the effect of Covid-19 and lockdown control measures on maternal and perinatal outcomes, a single-centre cross-sectional study at Mpilo Central Hospital. *BMC Pregnancy Childbirth*. 2021;21(1):416.

87. Shikuku DN, Nyaoke IK, Nyaga LN, Ameh CA. Early indirect impact of COVID-19 pandemic on utilisation and outcomes of reproductive, maternal, newborn, child and adolescent health services in Kenya: A cross-sectional study. *Afr J Reprod Health*. 2021;25(6):76-87.

88. Simeone RM, Downing KF, Wallace B, Galang RR, DeSisto CL, Tong VT, Zapata LB, et al. Changes in rates of adverse pregnancy outcomes during the COVID-19 pandemic: a cross-sectional study in the United States, 2019-2020. *J Perinatol*. 2022;42(5):617-623.

89. Simpson AN, Snelgrove JW, Sutradhar R, Everett K, Liu N, Baxter NN. Perinatal Outcomes During the COVID-19 Pandemic in Ontario, Canada. *JAMA Netw Open*. 2021;4(5):e2110104.

90. Son M, Gallagher K, Lo JY, Lindgren E, Burris HH, Dysart K, Greenspan J, et al. Coronavirus Disease 2019 (COVID-19) Pandemic and Pregnancy Outcomes in a U.S. Population. *Obstet Gynecol.* 2021;138(4):542-551.

91. Stowe J, Smith H, Thurland K, Ramsay ME, Andrews N, Ladhani SN. Stillbirths During the COVID-19 Pandemic in England, April-June 2020. *JAMA*. 2021;325(1):86-87.

92. Stumpfe FM, Schneider MO, Hein A, Faschingbauer F, Kehl S, Hermanek P, Böhm J, et al. Limited Effects of SARS-CoV-2 Pandemic-related Lockdowns and Reduced Population Mobility on Preterm Birth Rates: A Secondary Analysis of Bavarian Obstetric Quality Parameters from 2010 to 2020. *Geburtshilfe Frauenheilkd*. 2022;82(8):842-851.

93. Vani K, Estrada Trejo F, Plagianos M, Segui M, Vorawandthanachai T, Nathan L, Hughes F. Incidence and characteristics of stillbirths before and during the Coronavirus 2019 pandemic. *J Matern Fetal Neonatal Med*. 2022;35(26):10324-10329.

94. Wang B, An H, Hu H, Zhao W, Jiangtulu B, Wang S, Wang J, et al. Clinical Effects of the COVID-19 Pandemic Among the Uninfected Pregnant Women - 6 PLADs, China, 2019-2020. *China CDC Wkly*. 2021;3(10):199-206.

95. Wang J, Wang Y, He MY, Li YX, Cheng X, Yang X, Li RM, et al. Maternal and infant outcomes during the COVID-19 pandemic: a retrospective study in Guangzhou, China. *Reprod Biol Endocrinol*. 2021;19(1):126.

96. Wen J. Impact of COVID-19 pandemic on birth outcomes: A retrospective cohort study in Nanjing, China. *Front Public Health*. 2022;10:923324.

97. Wood R, Sinnott C, Goldfarb I, Clapp M, McElrath T, Little S. Preterm Birth During the Coronavirus Disease 2019 (COVID-19) Pandemic in a Large Hospital System in the United States. *Obstet Gynecol*. 2021;137(3):403-404.

98. Xie Y, Mu Y, Chen P, Liu Z, Wang Y, Li Q, Li M, Liang J, Zhu J. Interrupted-time-series analysis of the immediate impact of COVID-19 mitigation measures on preterm birth in China. *Nat Commun*. 2022;13(1):5190.

99. Yalçin SS, Boran P, Tezel B, Şahlar TE, Özdemir P, Keskinkiliç B, Kara F. Effects of the COVID-19 pandemic on perinatal outcomes: a retrospective cohort study from Turkey. *BMC Pregnancy Childbirth*. 2022;22(1):51.

100. Yildiz Y, Gurlek B, Yildiz İE, Aydin T, Kanburoglu MK, Yilmaz B. The effects of Coronavirus disease-2019 (COVID-19) pandemic on routine antenatal care visits and complications of pregnancy. *Rev Assoc Med Bras (1992).* 2021;67(6):833-838.
